# Genome-wide evolutionary dynamics of influenza B viruses on a global scale

**DOI:** 10.1371/journal.ppat.1006749

**Published:** 2017-12-28

**Authors:** Pinky Langat, Jayna Raghwani, Gytis Dudas, Thomas A. Bowden, Stephanie Edwards, Astrid Gall, Trevor Bedford, Andrew Rambaut, Rodney S. Daniels, Colin A. Russell, Oliver G. Pybus, John McCauley, Paul Kellam, Simon J. Watson

**Affiliations:** 1 Wellcome Trust Sanger Institute, Hinxton, United Kingdom; 2 Department of Zoology, University of Oxford, Oxford, United Kingdom; 3 Institute of Evolutionary Biology, University of Edinburgh, Edinburgh, United Kingdom; 4 Vaccine and Infectious Disease Division, Fred Hutchinson Cancer Research Center, Seattle, Washington, United States of America; 5 Division of Structural Biology, Wellcome Trust Centre for Human Genetics, University of Oxford, Oxford, United Kingdom; 6 Fogarty International Center, National Institutes of Health, Bethesda, Maryland, United States of America; 7 Worldwide Influenza Centre, The Francis Crick Institute, London, United Kingdom; 8 Department of Veterinary Medicine, University of Cambridge, Cambridge, United Kingdom; Katholieke Unversiteit Leuven, BELGIUM

## Abstract

The global-scale epidemiology and genome-wide evolutionary dynamics of influenza B remain poorly understood compared with influenza A viruses. We compiled a spatio-temporally comprehensive dataset of influenza B viruses, comprising over 2,500 genomes sampled worldwide between 1987 and 2015, including 382 newly-sequenced genomes that fill substantial gaps in previous molecular surveillance studies. Our contributed data increase the number of available influenza B virus genomes in Europe, Africa and Central Asia, improving the global context to study influenza B viruses. We reveal Yamagata-lineage diversity results from co-circulation of two antigenically-distinct groups that also segregate genetically across the entire genome, without evidence of intra-lineage reassortment. In contrast, Victoria-lineage diversity stems from geographic segregation of different genetic clades, with variability in the degree of geographic spread among clades. Differences between the lineages are reflected in their antigenic dynamics, as Yamagata-lineage viruses show alternating dominance between antigenic groups, while Victoria-lineage viruses show antigenic drift of a single lineage. Structural mapping of amino acid substitutions on trunk branches of influenza B gene phylogenies further supports these antigenic differences and highlights two potential mechanisms of adaptation for polymerase activity. Our study provides new insights into the epidemiological and molecular processes shaping influenza B virus evolution globally.

## Author summary

Influenza B viruses cause roughly one third of the global influenza disease burden. However, many important questions regarding the global-scale molecular epidemiology and evolutionary dynamics of influenza B virus have yet to be comprehensively addressed compared to influenza A virus. This is in part due to limited globally-sampled genomic data. We improved the availability of influenza B virus data by sequencing over 350 full genomes, fillings gaps from under-sampled regions by as much as 12-fold. Using a dataset of over 2,500 influenza B virus genomes, we show major differences in the genome-wide evolution, molecular adaptation, and geographic spread between the two major influenza B lineages. These findings have implications for vaccine design and improve our understanding of influenza virus evolution.

## Introduction

Influenza viruses cause significant morbidity and mortality worldwide and present major challenges for public health. Two types of influenza virus circulate widely in human populations: influenza A and influenza B viruses. While rates of hospitalization and mortality attributed to influenza B are lower than for influenza A subtype A(H3N2), they were higher than the less virulent seasonal A(H1N1) subtype of influenza A viruses [1]. Influenza B viruses cause epidemics worldwide each year, contributing approximately one third of the global influenza disease burden [2], and are associated particularly with severe disease in children [1,3]. Despite the significance of influenza B viruses to public health, their epidemiological characteristics and their global evolutionary and antigenic dynamics are poorly understood compared to influenza A viruses [4,5]. Influenza B viruses are classified into two co-circulating phylogenetically- and antigenically-distinct lineages, named after viruses B/Yamagata/16/88 (Yamagata-lineage) and B/Victoria/2/87 (Victoria-lineage) that diverged in the 1970s [6,7]. The Yamagata- and Victoria-lineages have had a complex epidemiological history since their divergence, co-circulating globally since at least 2002 and often alternating in regional dominance [8]. Disparities from antigenic mismatches between the predominant circulating influenza B virus lineage in a given year and that year’s seasonal influenza trivalent vaccine (which contains representatives of A(H1N1), A(H3N2) plus one of the two influenza B virus lineages) have occurred. Consequently, updated quadrivalent vaccines that contain representative Yamagata-lineage and Victoria-lineage viruses have been recommended [9].

A number of studies have reported the genetic and epidemiological characteristics of influenza B viruses in specific geographic regions [2,10–15] yet few have investigated the large-scale evolutionary dynamics of influenza B viruses at the genome-wide level or global scale [16–19]. Nevertheless, existing insights into the evolutionary dynamics of influenza B viruses show they undergo slower antigenic evolution than influenza A viruses [19,20], with genetic changes including nucleotide insertions, nucleotide deletions, and frequent reassortment events between and within lineages, contributing to their continued diversification [16,17,21,22]. Recent analyses have revealed that the polymerase basic 1 and 2 (PB1, PB2) and hemagglutinin (HA) genes of Victoria- and Yamagata-lineage viruses remain as distinct lineages despite high levels of overall reassortment, likely through genomic incompatibility among viral genome segments [17,23]. Other differences between the two lineages have been observed; Victoria-lineage viruses appear to undergo more rapid lineage turnover and antigenic drift [18] and persist for longer in local geographic regions before wider dissemination [19]. Despite these advances, there remain substantial unanswered questions about the genomic evolution of influenza B viruses on a global scale, including whether the genetic differentiation observed in HA is mirrored in other less-studied gene segments and the influence of geography on genome-wide viral genetic diversity. Until recently, efforts to address these issues have been hampered by the paucity of globally sampled influenza B virus hemagglutination inhibition (HI) data and full-length genome sequences available, particularly from Europe, Africa, Central Asia, and South America.

To address this, we used samples from multiple locations worldwide to generate 382 new complete influenza B virus genome sequences. We further compiled the largest and most spatio-temporally-representative dataset of influenza B virus whole genome sequences to date. This dataset included 2,651 complete genomes (1,265 Yamagata- and 1,386 Victoria-lineage HA viruses) sampled worldwide between 1987 and 2015. We used antigenic cartography and phylogenetic approaches to identify patterns of reassortment, compare the dynamics of antigenic evolution among lineages, and characterize genome-wide demographic histories in geographic regions. We identify substitutions along the trunk branches of the phylogenies for each gene and structurally map changes in the HA and polymerase complex that may contribute to molecular adaptation. Our study shows how the global phylodynamics and epidemiologic interactions of influenza B viruses are shaped by reassortment, genomic compatibility, and differing patterns of antigenic change.

## Results

### New influenza B virus genome sequences from multiple locations worldwide

**Fig 1:**
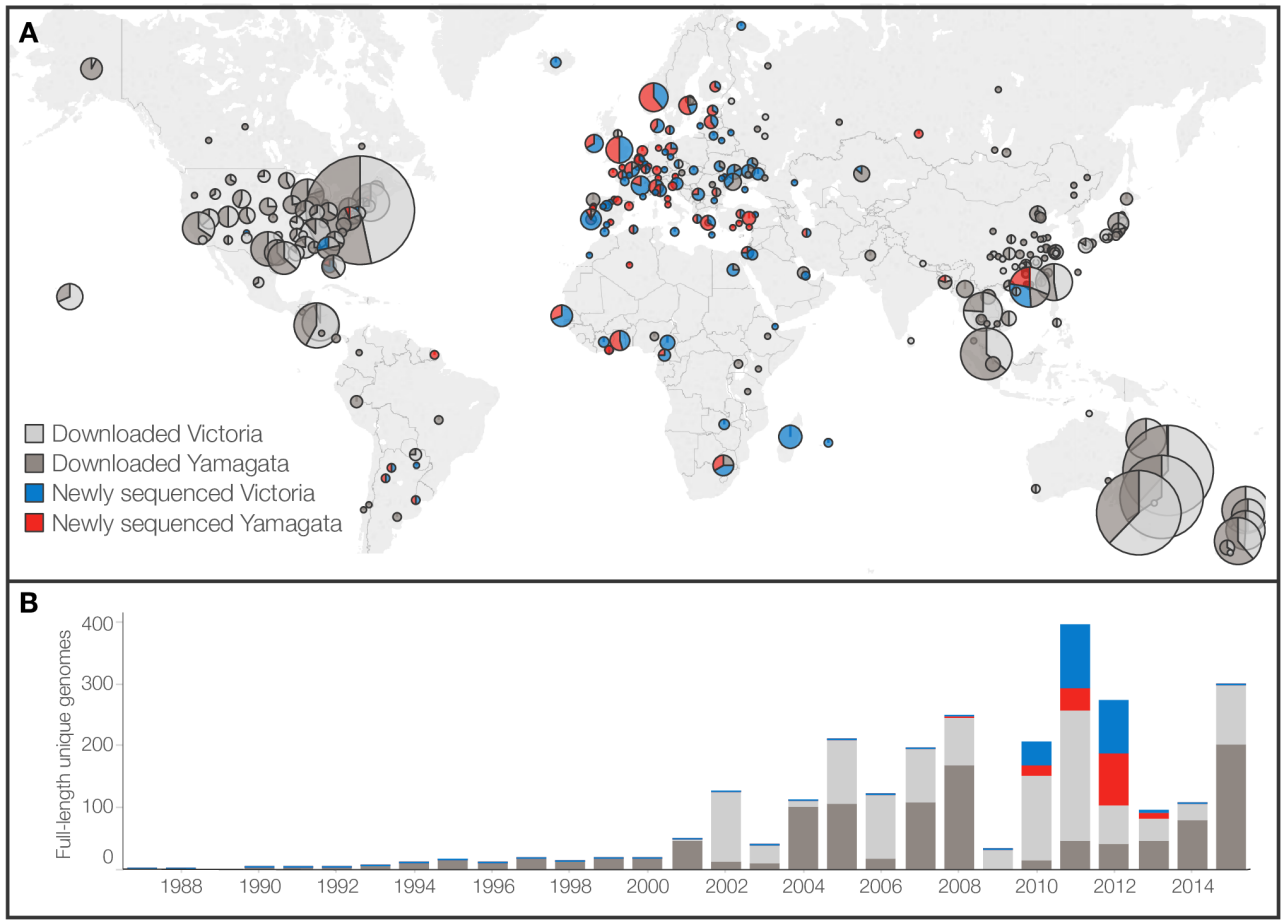
Source and distribution of available influenza B virus full genomes. (A) Geographic source and (B) temporal distribution of 2,651 unique complete genomes analyzed in this study. Circle areas are proportional to the number of unique viruses originating from a location; smallest circle size represents 1 genome, largest circle size represents 332 genomes. Pie chart fractions reflect proportion of unique full genomes that were either newly generated in this study or downloaded from IVR and GISAID (on 25 August 2015). Viruses are classified as Victoria- or Yamagata-lineage by HA gene.

For this study, we sequenced and assembled 382 new, full-length genomes of influenza B viruses collected globally from 2007 to 2013 (Fig 1). In total, we analyzed all available gene sequence data from over 10,000 distinct influenza B viruses sampled from 1987 to 2015, of which 2,651 were complete genomes. Our sequencing efforts increased the total number of complete influenza B genomes by 17%, with the new genomes representing a 44% increase in the number of genomes for the years 2008–2013 (Fig 1B). Crucially, our genomes were sampled from geographic regions under-represented by previous influenza B virus molecular surveillance. Specifically, we increased the number of genomes from Europe (20 to 243 genomes), Africa (11 to 89 genomes), Central Asia (10 to 37 genomes), and South America (21 to 31 genomes). Our sequencing has therefore substantially improved the global context of influenza B genomic diversity (Fig 1A). One region that remains deficient in influenza B genome sequences is the Indian subcontinent, as assessed by lack of submission to sequence databases, which was previously shown to be an important source of influenza A and B virus diversity [19]. Despite this, our study encompasses the most comprehensive dataset of influenza B complete genomes to date.

### Divergence and reassortment in Yamagata- and Victoria-lineage viruses

**Fig 2:**
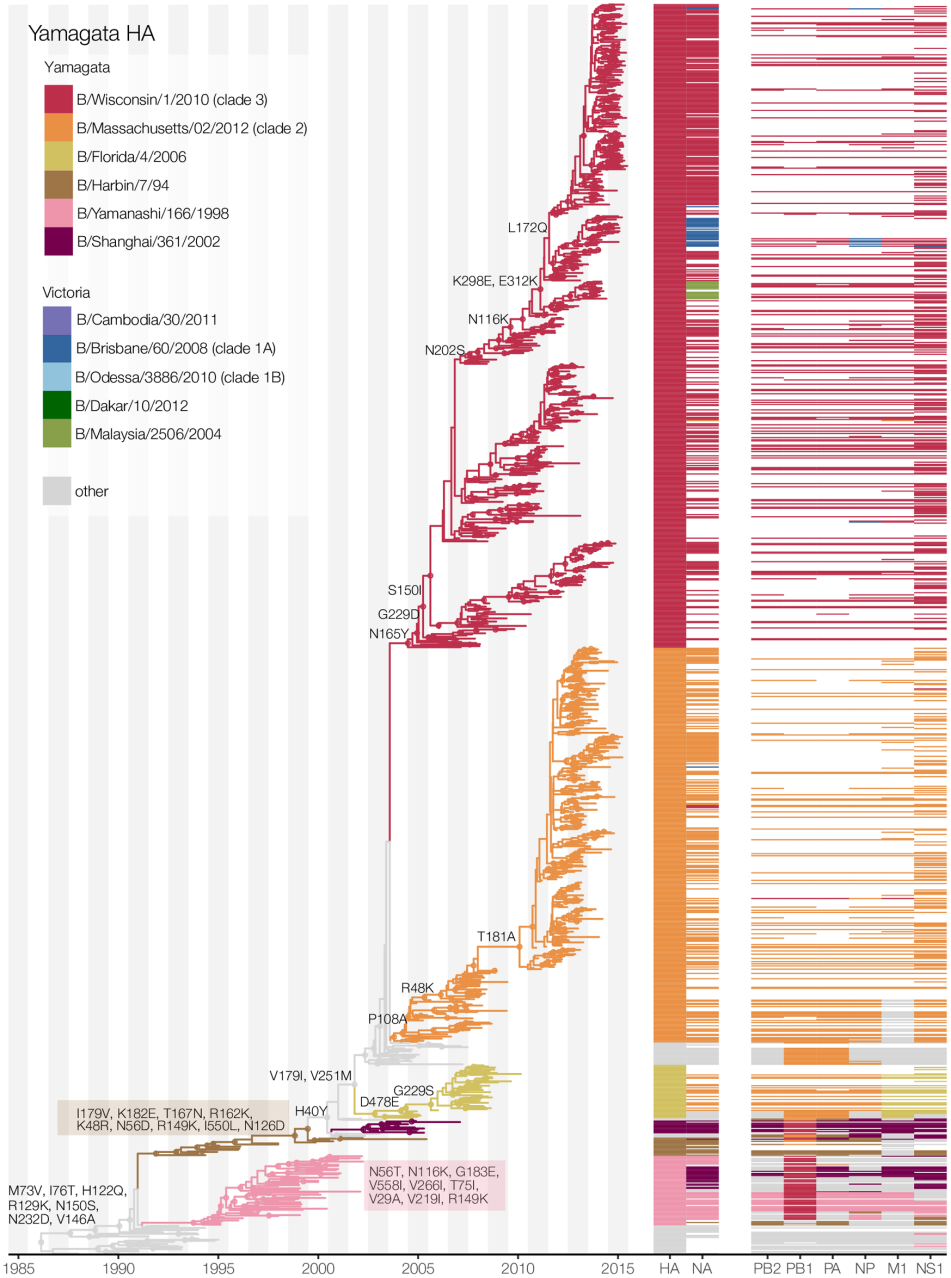
Maximum clade credibility tree inferred from 1,169 Yamagata-lineage HA gene sequences and corresponding genotype constellations. Branches of the phylogeny are labeled with amino acid substitutions occurring along the phylogenetic ‘trunk’ and colored by well-supported clade distinction (see legend). Clade classifications of each gene are similarly indicated by colored bars. White bars indicate that no sequence was available for that gene. Nodes with greater than 0.70 posterior probability support are shown with circle node shapes.

**Fig 3:**
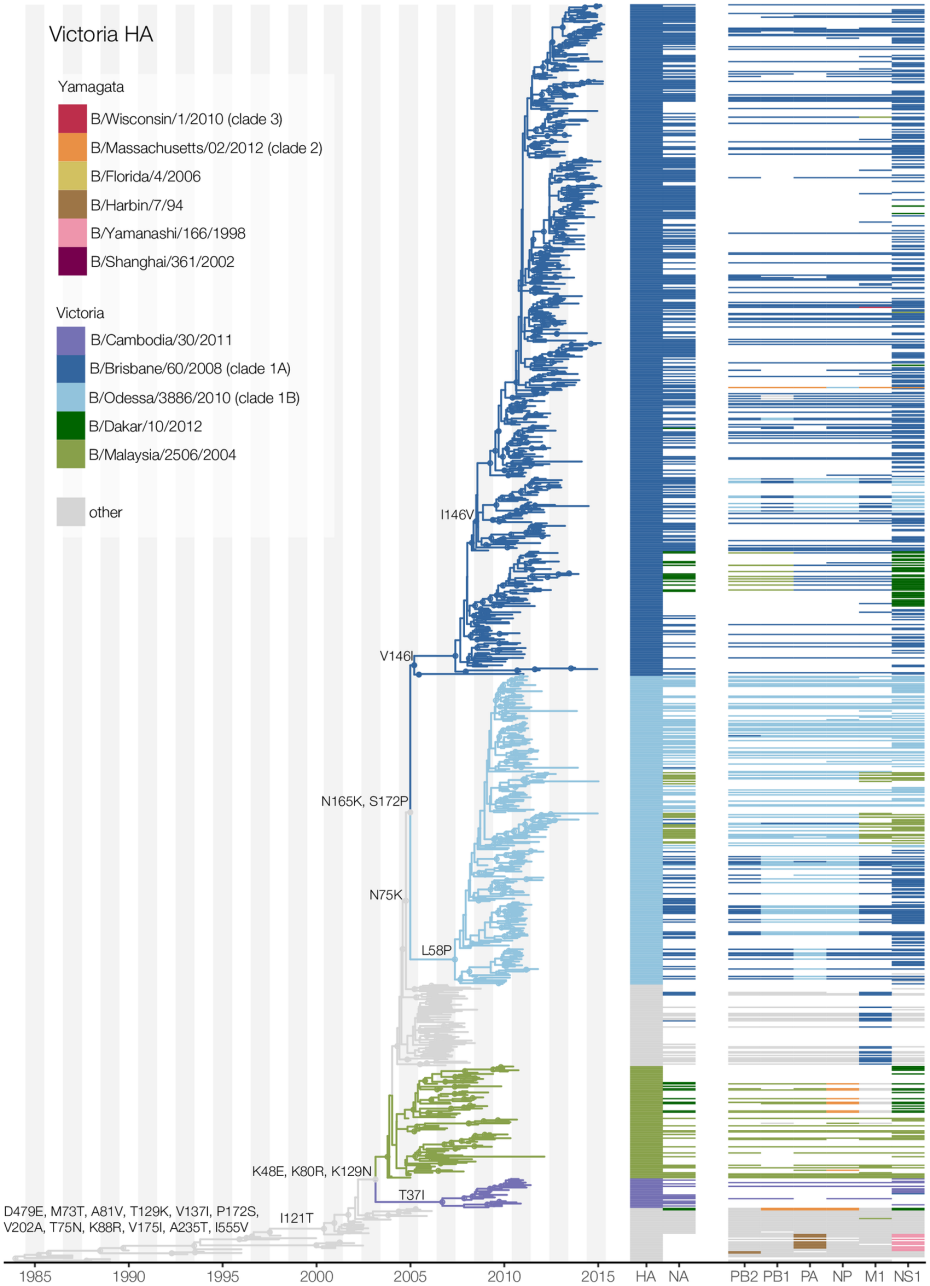
Maximum clade credibility tree inferred from 1,019 Victoria-lineage HA gene sequences and corresponding genotype constellations. See legend to Fig 2 for details.

The Yamagata-lineage has been separated previously into two major antigenically distinct clades (clade 2, the B/Massachusetts/02/2012 clade, and clade 3, the B/Wisconsin/1/2010 clade), based on phylogenetic analysis of its HA and neuraminidase (NA) genes [24,25]. However, it was unknown whether this separation also extended to the other genes. Our analysis demonstrates that this phylogenetic divergence is indeed present across all genes, resulting in each Yamagata-lineage clade comprising a distinct ‘whole genome’ genotype (Fig 2, S1 Fig). Using molecular clock phylogenetic analysis, we estimated that this whole genome split occurred progressively over a period of approximately 10 years, beginning with the PB1 segment around 1993 (95% highest posterior density (HPD) 1992–1994) (S2 Fig), followed by polymerase acidic protein (PA) in 1996 (95% HPD 1995–1997), then nucleoprotein (NP), PB2, HA, NA, non-structural protein 1 (NS1), and matrix protein 1 (M1) in 2002–2003 (95% HPD 2001–2004) (Table 1). While several Yamagata-Victoria inter-lineage reassortment events were apparent after the genome-wide split of Yamagata-lineage viruses into clades 2 and 3, especially for NA, we observe that after the split of Yamagata-lineage viruses, there is little evidence of substantial reassortment between the Yamagata-lineage clades, with them maintaining their unique genomic constellations for over 12 years (Fig 2, S2 Fig). In contrast, Victoria-lineage influenza B viruses show evidence of continued reassortment between clades within the Victoria-lineage over time. As a result, we observed multiple co-circulating Victoria clades that do not maintain distinct genome constellations (Fig 3, S1 and S3 Figs). In particular, we noted considerable inter-clade reassortment between recently circulating B/Brisbane/60/2008 (clade 1A), B/Odessa/3886/2010 (clade 1B), and B/Malaysia/2506/2004 clade viruses.

**Table 1:** Estimated time of most recent common ancestor (TMRCA) for Yamagata-lineage clade 2 and clade 3 viruses.

Gene	Mean TMRCA	95% HPD lower	95% HPD upper
PB1	1993.690	1992.859	1994.292
PA	1996.780	1995.979	1997.474
NP	2002.545	2001.975	2002.995
PB2	2003.070	2002.727	2003.537
NA	2003.100	2002.443	2003.743
HA	2003.240	2002.831	2003.789
NS1	2003.500	2002.942	2003.974
M1	2003.550	2002.628	2004.980

### Dynamics of antigenic evolution differ between Victoria- and Yamagata-lineage viruses

**Fig 4:**
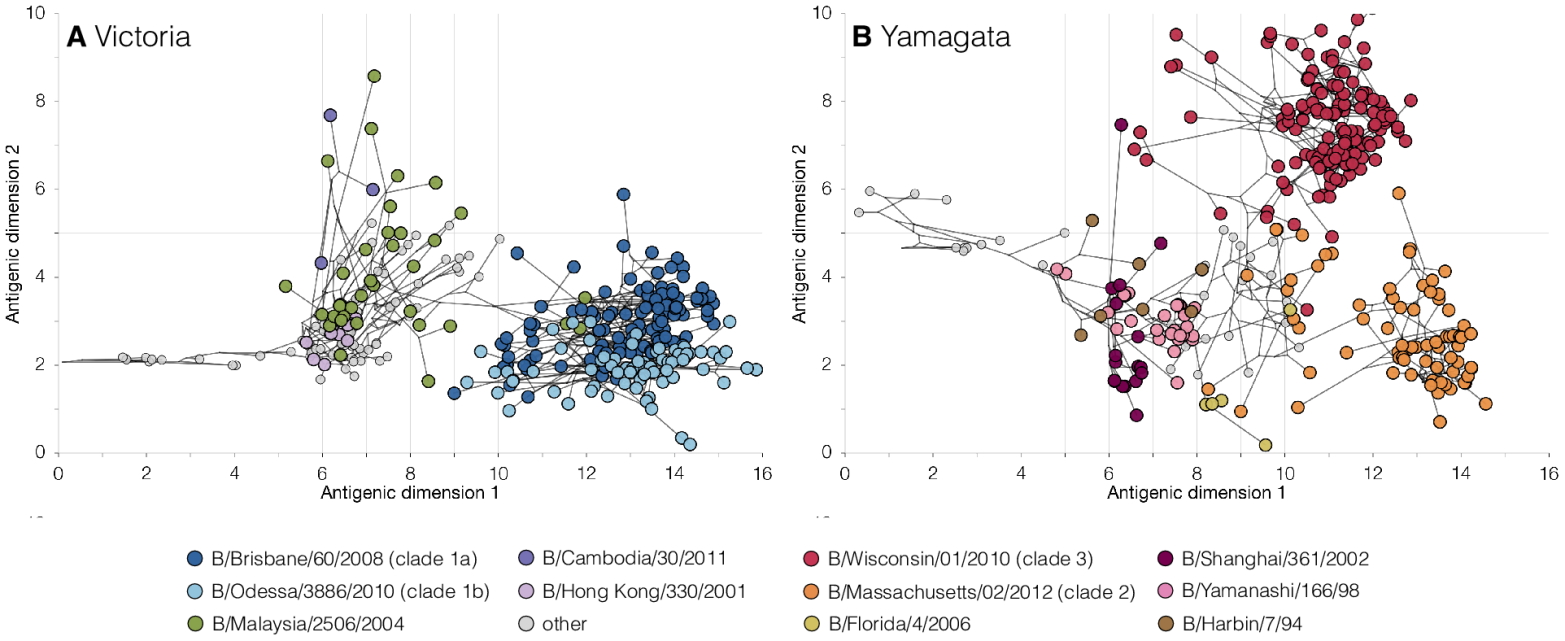
Antigenic and genetic evolutionary relationships of influenza B viruses. Antigenic maps of (A) 309 Victoria- and (B) 308 Yamagata-lineage viruses shown in 2 antigenic dimensions over time (1987–2013), inferred by BMDS using HI titer data and HA sequences. Each circle indicates a virus antigenic map location and lines represent phylogenetic relationships inferred between viruses.

The abovementioned differences in the genome-wide evolutionary patterns between Yamagata- and Victoria-lineage viruses led us to investigate if the genetic differences also extended to the antigenic properties of the viruses, as measured by hemagglutination inhibition (HI) data. We compiled available HI measurements and associated HA gene sequences for influenza B viruses sampled between 1987–2013. We then removed known egg-adapted viruses, resulting in a dataset of 309 Victoria- and 308 Yamagata-lineage viruses with both genetic and antigenic data. We integrated these data under a Bayesian framework [20] to jointly infer the antigenic and genetic relationships of influenza B viruses in two antigenic dimensions (Fig 4, S4 Fig). Under a Bayesian multidimensional scaling (BMDS) model that does not account for variations in virus avidities and serum potencies in the HI assays (‘fixed effects,’ model 7 in [20]), the two extant Yamagata-lineage clades appear to experience little antigenic change over time (S4 and S5 Figs) with an estimated drift rate slower than the Victoria-lineage, in line with previous observations by Vijaykrishna *et al*. [18]. However, using a more comprehensive model that does consider these experimental variations (‘full model,’ model 10 in [20]), we found no significant difference in antigenic drift rate between the Victoria-lineage and the Yamagata-lineage (Table 2), in agreement with Bedford *et al*. [20]. Previous model performance testing indicated that the latter model provided the greatest predictive power and least test error for HI titers [20], providing further support for influenza B virus lineages experiencing antigenic drift at similar rates.

**Table 2:** Estimated antigenic drift rate for influenza B virus Victoria and Yamagata lineages inferred using BMDS.

	Full model		Fixed effects model	
	Victoria	Yamagata	Victoria	Yamagata
**Antigenic drift rate (AU/year)**	0.37	0.34	0.32	0.12
**95% HPD (AU/year)**	0.25–0.47	0.22–0.45	0.23–0.39	0.03–0.21

Despite comparable rates of antigenic drift, we observed notable differences in the dynamics of antigenic evolution between the Victoria- and Yamagata- lineages. Around 2005, the genetically-distinct clades 1A and 1B of the Victoria-lineage emerged, replacing the previously-circulating lineages and subsequently dominating the Victoria-lineage virus population (Fig 3). While the HA genes of these Victoria-lineage clades are clearly different (Fig 3), antigenic mapping showed they are not antigenically distinct (Fig 4A). Conversely, the genetically-divergent Yamagata-lineage clade 2 and 3 viruses do exhibit measurable antigenic divergence (Fig 4B). In contrast to the serial replacement of novel antigenic types in the Victoria-lineage viruses (Fig 4A), the two antigenically-distinct clades of the Yamagata-lineage co-circulate globally, alternating in dominance (nextflu.org/yam/12y/) (S6 Fig). However, despite the divergence and counter-cyclical maintenance of Yamagata-lineage clades 2 and 3 over 10 years, recent reports indicate that the incidence of clade 2 viruses has decreased substantially (https://www.crick.ac.uk/research/worldwide-influenza-centre/annual-and-interim-reports/).

Other long-lived Yamagata-lineage clades previously became extinct. In particular, B/Yamanashi/166/98 clade viruses emerged in 1993 (95% HPD: 1992–1994), and constituted the predominant circulating Yamagata-lineage clade worldwide until 2002, when they were replaced by B/Harbin/7/94-like Yamagata-lineage viruses (Fig 2). Although these two Yamagata-lineage clades were genetically distinct, the B/Harbin/7/94 clade was antigenically similar to the B/Yamanashi/166/98 clade (Fig 4B). Our whole-genome phylogenetic analysis showed that in 2000–2001 (95% HPD: April 2000-April 2001) the B/Yamanashi/166/98 clade provided the NA gene that became incorporated into the Victoria-lineage (S3 Fig). Subsequently, the global incidence of Victoria-lineage viruses increased dramatically, while the B/Yamanashi/166/98 clade went extinct. This suggests that factors involving other gene segments or differing patterns of reassortment may have influenced influenza B lineage dynamics on a global scale. However, we were unable to investigate this further due to limited availability of genome sequences covering this time period.

### Structural mapping of phylogenetic ‘trunk’ nonsynonymous substitutions

**Fig 5:**
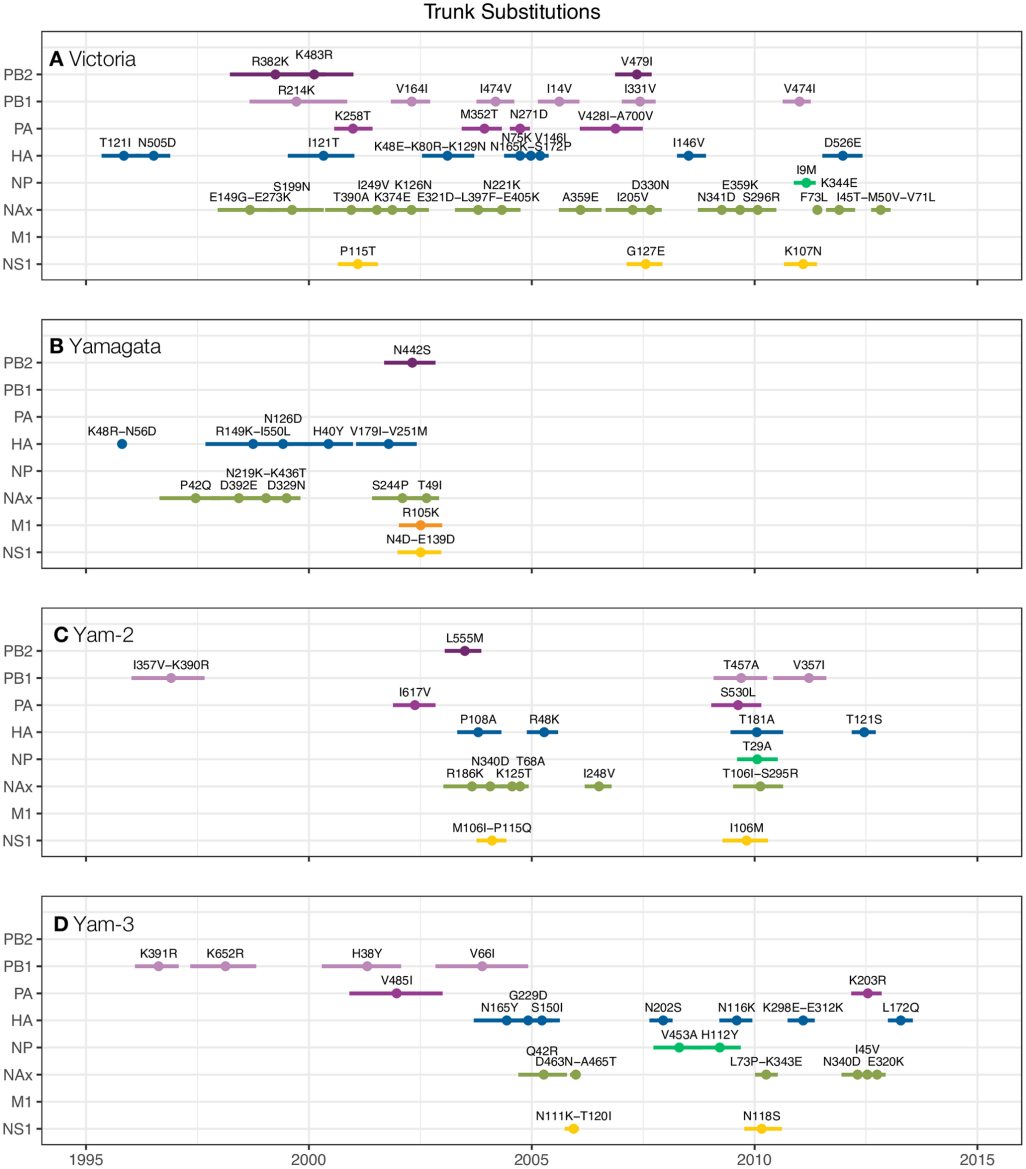
Estimated emergence of nonsynonymous substitutions along the major trunk lineage of the gene phylogenies of Victoria- and Yamagata-lineage virus. Substitutions are summarized from S1 Fig for (A) Victoria-lineage, (B) Yamagata-lineage, (C) Yamagata-lineage clade 2 (B/Massachusetts/02/2012 clade), and (D) Yamagata-lineage clade 3 (B/Wisconsin/1/2010 clade), with only substitutions emerging after 1995 shown for clarity. Circles represent median and lines represent 95% HPD estimates of time of emergence across 1,000 posterior trees.

Given the observed influenza B virus inter-lineage differences in the phylodynamics and patterns of antigenic evolution, we sought to compare levels of natural selection acting on Victoria- and Yamagata-lineage viruses. As selective sweeps are difficult to detect by dN/dS methods, we used ancestral sequence reconstruction to quantify the accumulation of potentially adaptive substitutions in all the major influenza B virus genes (Fig 5, S1 Fig, S1 Table). We focused on amino acid changes occurring on the ‘trunk’ of the phylogenies, which are less sensitive to varying sampling densities over time that occur due to differences in sequence availability. Substitutions along the trunk represent changes that have fixed in the virus population and are at least neutral or could confer selective advantages that are swept to fixation. We first compared trunk substitutions in Victoria- and Yamagata- lineage HA phylogenies (S7 Fig). Fewer nonsynonymous changes were found along the trunk of the Victoria-lineage HA phylogeny (mean: 0.81; 95% HPD: 0.76–0.86 nonsynonymous substitutions/year) than the Yamagata-lineage phylogeny (mean: 1.06; 95% HPD: 0.93–1.17 nonsynonymous substitutions/year) (Figs 2 and 3). Structural mapping of these trunk mutations showed that, in both lineages, the majority of changes were in solvent-accessible residues on the globular head region of HA (S7 and S8 Figs). As expected, these substitutions predominantly occurred within predicted antigenic epitopes in the Yamagata- and Victoria-lineages [26, 27] (S7 and S8 Figs).

Since 2002 and the global re-emergence of the Victoria-lineage [16], both lineages have experienced trunk substitutions in three residues located in HA1 antigenic epitopes; Yamagata-lineage (amino acid changes N116K, S150I, N202S) and Victoria-lineage (amino acid changes K129N, I146V, N165K) (Figs 2 and 3, S7 Fig). Previous experimental work has shown that transitions between influenza antigenic clusters are predominantly associated with substitutions at sites near the receptor-binding site (RBS) [28]. We identified four trunk substitutions adjacent to the RBS: V137I, which fixed early in Victoria-lineage HA prior to 1995 (Fig 3); residues N150S (and S150N), R162K, and N202S in Yamagata-lineage HA. We identified a smaller number of trunk substitutions in structurally ‘buried’ residues, namely P108S, V179I and V25M1 in Yamagata-lineage HA, with P108A notably a clade 2-defining substitution that fixed early in the Yamagata-lineage clade 2/3 divergence (Fig 2).

Ancestral sequence reconstruction along the Victoria- and Yamagata-lineage HA phylogenies also revealed residues that experienced multiple amino acid replacement and therefore temporary fixation over time (Figs 2 and 3; S2 Table). For Victoria-lineage viruses, two such positions (T75N/N75K, T129K/K129N) were solvent-accessible (exposed) residues of known antigenic epitopes. For Yamagata-lineage viruses, residue N150S/S150I was in a partially-exposed position within a major antigenic epitope, adjacent to the RBS. Additionally, we observed a number of residues that experienced amino acid substitutions that subsequently reverted back to their ancestral state. Three of these HA reversions (K48R/R48K in Yamagata-lineage, V146I/I146V and T121I/I121T in Victoria-lineage) occurred in known antigenic epitopes, while two other reversion residues (172 in Victoria- and 179 in Yamagata-lineages) were not located in or near predicted epitopes (S2 Table). Furthermore, we observed identical substitutions in major antigenic epitopes (N116K, R149K) that emerged and became independently fixed in different Yamagata-lineage clades. Both substitutions occurred in the B/Yamanashi/166/98 clade that went extinct around 2002, and around a similar time, R149K also arose in B/Harbin/7/94 viruses. More recently, N116K became fixed in clade 3 viruses. Finally we observed changes to a given residue that were different depending on the Yamagata-lineage clade. In particular, around the year 2005, changes at residue 229 were independently fixed in Yamagata-lineage clade 3 (as G229D) and clade 1 (B/Florida/4/2006 clade: G229S); clade 2 Yamagata-lineage viruses however, retained the ancestral amino acid (229G) at this site. Consequently, from 2005–2010, the Yamagata-lineage comprised three co-circulating populations that varied at position 229 in HA.

**Fig 6:**
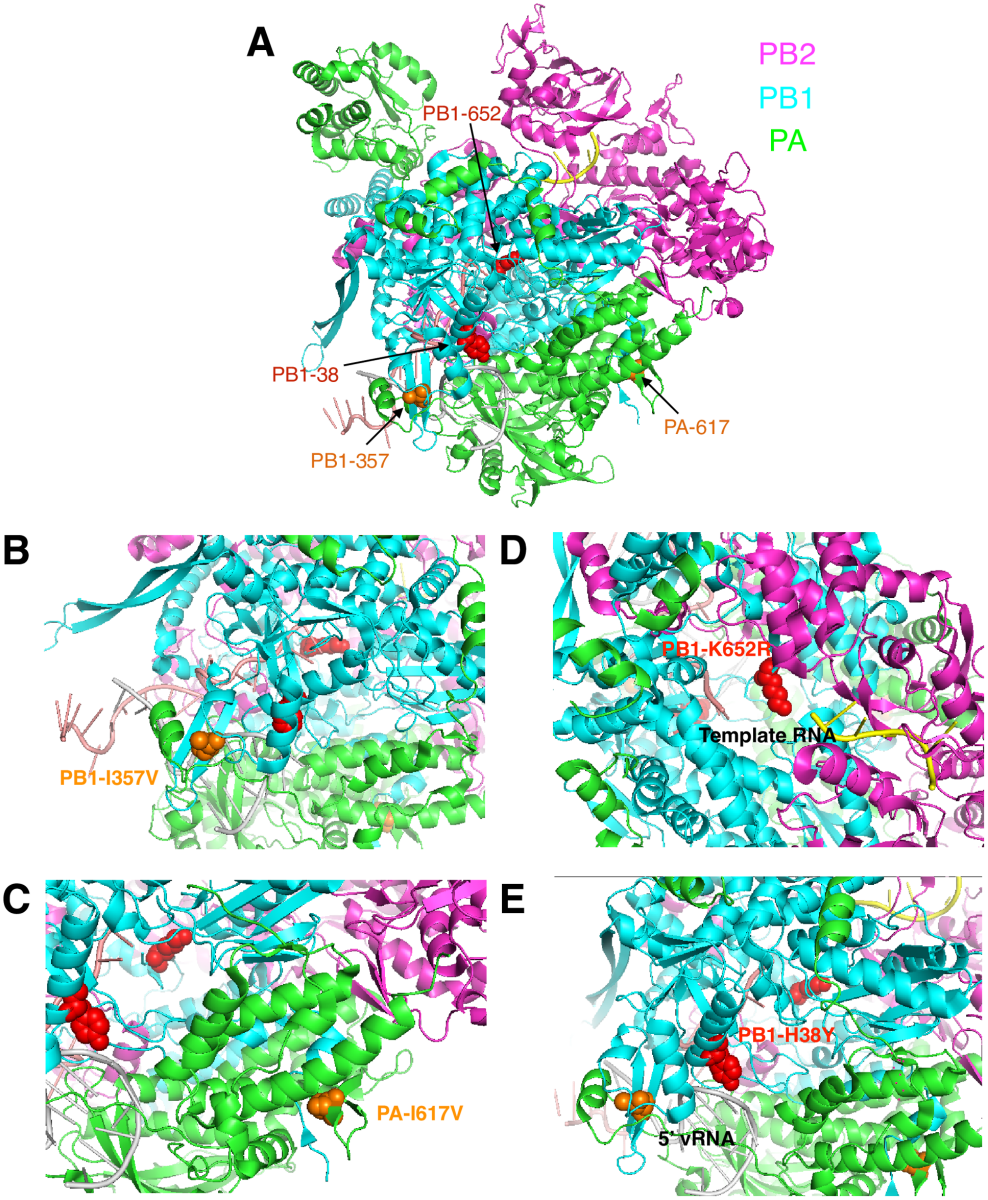
Structural mapping of major Yamagata-lineage ‘clade-defining’ trunk substitutions on influenza B virus polymerase complex. (A) Crystal structure of influenza B virus polymerase (PDB: 5MSG) bound to template vRNA shown in cartoon view colored by subunit, highlighting locations of residues of interest as spheres colored by presence of change in Yamagata-lineage clade 2 (orange) or clade 3 (red) viruses. Zoomed-in view of substitutions (B, C) at PB1-PA interface fixed in clade 2 viruses and (D, E) at PB1-vRNA interface fixed in clade 3 viruses.

Applying the same rationale, we estimated the time of emergence of trunk substitutions across the entire genome of Victoria- and Yamagata-lineage viruses (Fig 5). Over the 20-year period, only one amino acid change, R105K present in contemporary Yamagata-lineage viruses of both clades, fixed in matrix protein (M1) in the global influenza B virus population (Fig 5B, S1B Fig). There was potential co-emergence of substitutions in some gene segments, for example emergence of trunk substitutions in NS1 appeared to coincide with the emergence of substitutions in NA. There was also evidence of temporal ordering of Yamagata-lineage ‘clade-defining’ mutations, which first accumulated in PB1, followed by PA, and then the rest of the genes (Fig 5C and 5D). To determine whether these early trunk substitutions had potential functional consequences contributing to the clade 2/3 divergence of the Yamagata-lineage, we mapped them onto an influenza B virus polymerase complex structure (Fig 6). Yamagata-lineage clade 2 and clade 3 viruses accumulated changes in sites where PB1 and PA interact or where polymerase contacts viral RNA (vRNA), respectively. PB1-I357V and PA-I617V substitutions fixed in clade 2 viruses; both residues are positioned at the PB1-PA interface, with PA-617 at a known interaction site with the N-terminus of PB1 critical for PB1-PA binding [29] (Fig 6A and 6B). Differently, PB1-K652R and PB1-H38Y substitutions fixed in clade 3 viruses, both potentially interact with vRNA bound in the polymerase structure [30] (Fig 6C and 6D). Additional substitutions occurred in sites of the polymerase structure not at these interfaces. Around the same time (1996, 95% HPD 1996–1997 (S1 Table)), K390R and K391R PB1 substitutions emerged in Yamagata-lineage clade 2 and clade 3 viruses, respectively, which are located beside each other and are exposed on the polymerase structure (S9 Fig). Further, three ‘clade-defining’ substitutions that emerged later appeared to be ‘buried’ in the polymerase subunits: PB2-L555M in clade 2 viruses, and PA-V485I and PB1-V66I in clade 3 viruses (S9 Fig).

### Spatial population structure observed in Victoria- but not Yamagata-lineage viruses

Finally, we sought to determine whether the differences in the molecular evolutionary dynamics of Victoria- and Yamagata-lineage viruses that we observed at the global level were also present at regional scales. Previous studies have focused either on the circulation of influenza B viruses in a specific geographic region [18,31], or have analyzed the global circulation of the HA segment only. Unlike influenza A(H3N2) HA, influenza B HA lineages circulate independently in China, India, and Southeast Asia for long periods of time before spreading elsewhere in the world [19]. Here new data, especially from Europe, enables us to combine these two approaches and analyze whole virus genomes within specific geographic regions: Europe, the United States (USA), Australia and New Zealand (Oceania), and Southern China and Southeast Asia (SC/SEA).

**Fig 7:**
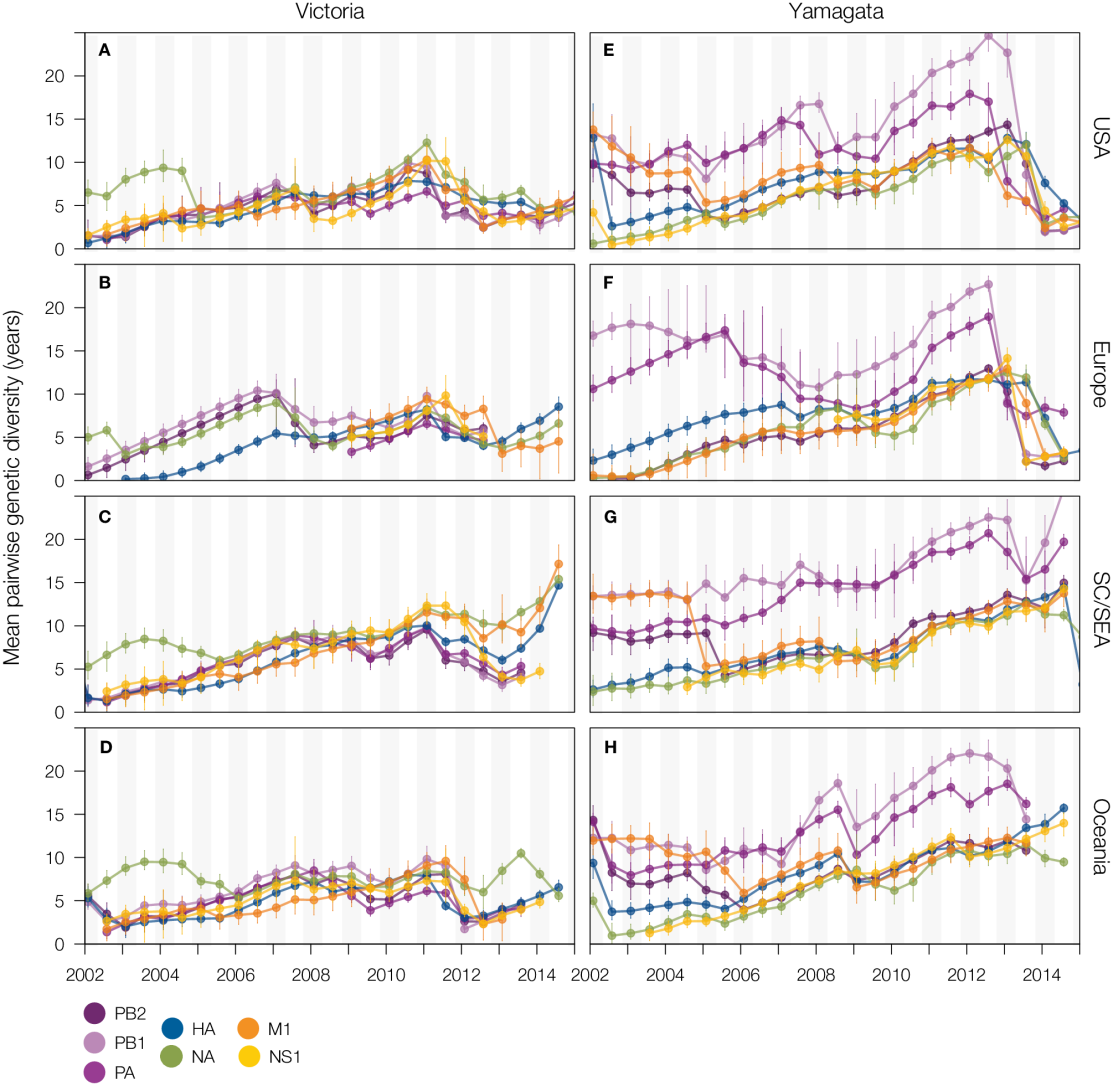
Genetic diversity of gene segments over time in different geographic regions. Time series of mean pairwise diversity (i.e. estimated average branch length distance between points in phylogeny at half- year intervals, measured in years) of Victoria- and Yamagata-lineage gene segments, for viruses collected from (A, E) USA, (B, F) Europe, (C, G) Southern China and Southeast Asia, and (D, H) Oceania.

**Fig 8:**
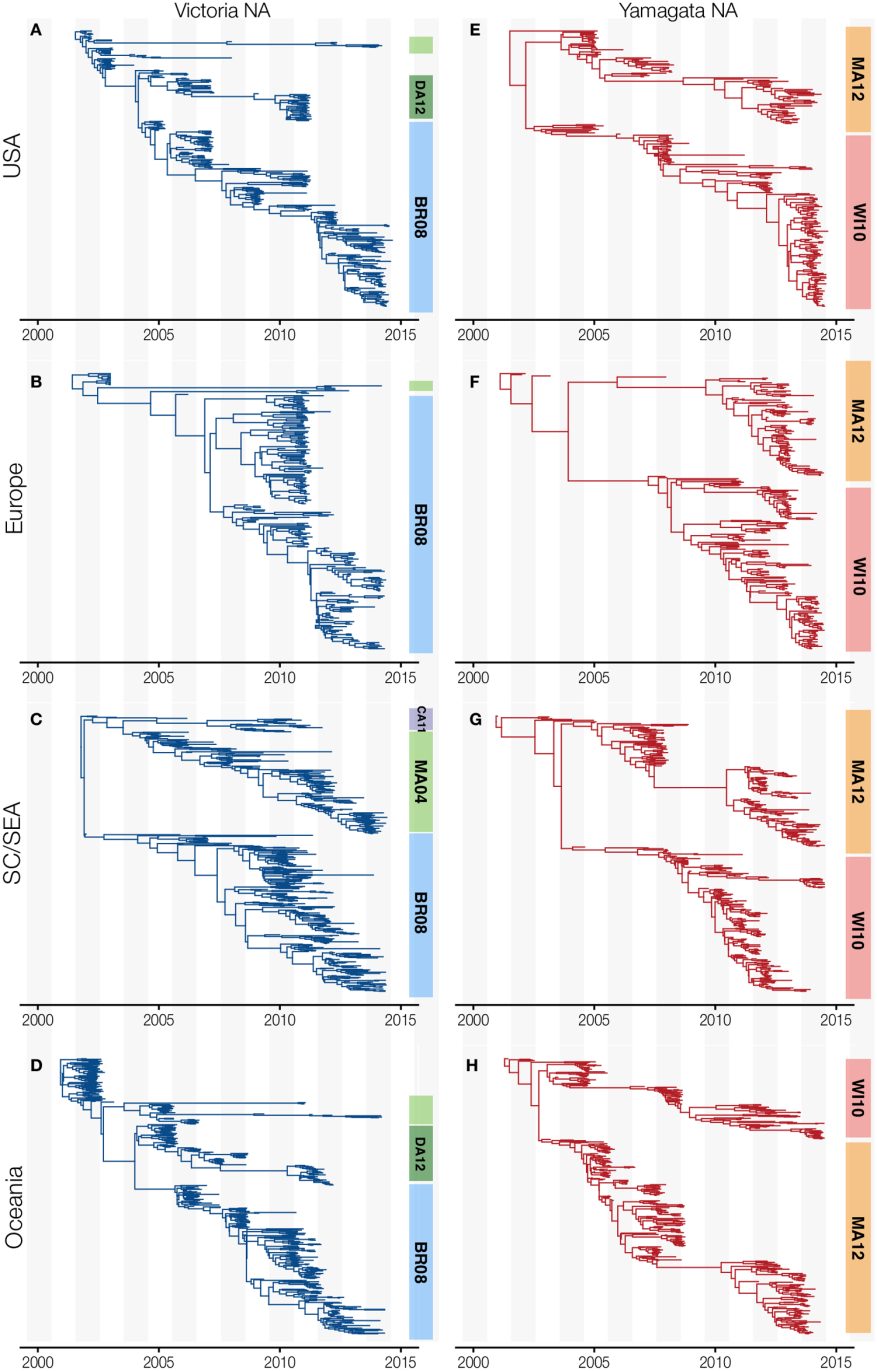
Time-resolved NA gene phylogenies of influenza B viruses isolated in four global regions from 2001–2014. Maximum-clade credibility (MCC) trees are shown for Victoria- (blue) and Yamagata-lineages (red) circulating in (A, E) USA, (B, F) Europe, (C, G) Southern China and Southeast Asia, and (D, H) Oceania. Clades are highlighted in colored blocks, Victoria-lineage: B/Brisbane/60/2008 clade (clade 1A; BR08) in blue, B/Malaysia/2506/2004 (MA04) in light green, B/Cambodia/30/2011 (CA11) in purple and B/Dakar/10/2012 (DA12) in dark green. For clarity, B/Odessa/3886/2010 clade (clade 1B) is shown as part of clade 1A (BR08). Yamagata-lineage: B/Massachusetts/2/2012 (clade 2; MA12) in orange and B/Wisconsin/1/2010 (clade 3; WI10) in light red.

Until 2011, Victoria-lineage viruses experienced selective sweeps across all segments simultaneously in different regions of the world (Fig 7). However, after 2011 regional differences became apparent, with only viruses in the USA and Europe maintaining this genome segment linkage (Fig 7A and 7B) whilst acquisition of the Victoria-lineage NA by Yamagata-lineage viruses in Oceania resulted in NA disassociating from the rest of the Victoria-lineage genome (Figs 7D and 8D, S10 Fig). Regional phylogenies also highlight the persistence of a Victoria-lineage NA gene (B/Malaysia/2506/2004 clade) that circulated almost exclusively within SC/SEA since 2003 (Fig 8C, S10 Fig). Throughout this period, viruses from this lineage were sporadically observed in other regions (Fig 8A, 8B and 8D), but did not persist outside of SC/SEA. Victoria-lineage viruses in SC/SEA show greater levels of inter- and intra-lineage reassortment, maintaining genetic diversity in NA, M1, and HA (Fig 7C). Unlike Victoria-lineage viruses, no major regional differences in the dynamics of genomic diversity were observed for the Yamagata-lineage (Fig 7E, 7F, 7G and 7H). Rather, the accumulation of diversity was associated with the split of the Yamagata-lineage into clades 2 and 3, with PB1 and PA showing greater accumulation of genetic diversity over time than other genes. Although influenza B virus sampling was more limited, these patterns of Victoria-lineage and Yamagata-lineage virus diversity were also observed for the geographic regions of Africa and the Eastern Mediterranean (S12 and S13 Figs).

Whole genome analysis of Victoria-lineage B/Malaysia/2506/2004 clade viruses revealed that they maintained a distinct genomic constellation until 2008–2009, when they underwent extensive reassortment of all segments except the NA gene (Fig 3, S3 Fig). The first reassortment event involved replacement of the HA, PB2, PB1, PA, and NP genes (95% HPD: March 2008-May 2009) with those from a globally co-circulating Victoria-lineage clade, the B/Odessa/3886/2010 1B clade. Following this, a subset of clade 3 viruses of the Yamagata-lineage that circulated in multiple geographic regions acquired the Victoria-lineage B/Malaysia/2506/2004 clade NA (95% HPD: June 2011-March 2012) (Fig 2). In a separate reassortment event, other viruses of the same Yamagata-lineage clade acquired the NA of the Victoria-lineage B/Brisbane/60/2008 1A clade, indicating a propensity for this Yamagata-lineage clade to replace its NA gene. However, despite this extensive reassortment, viruses containing the B/Malaysia/2506/2004-like NA gene are rarely detected outside of SC/SEA. Conversely, a B/Dakar/10/2012-like NA clade reassortant was observed in many regions of the world, but not in SC/SEA.

As influenza A viruses are known to exhibit different dynamics of lineage turnover among regions of the world [32], we decided to compare lineage turnover of influenza B viruses circulating in different geographic regions. To evaluate lineage turnover, we estimated the average time to most recent common ancestor (TMRCA) of contemporaneous viruses at yearly time intervals across the time-scaled phylogenies, which provides a measure of the maximum co-circulating genetic diversity in each year. For Victoria-lineage viruses from 2002–2015, the average estimated TMRCA is comparable in temperate regions with 4.1 years (3.6–4.7 years) in the USA, 4.1 years (3.7–4.8 years) in Europe, and 3.9 years (3.4–4.4 years) in Oceania. In comparison, the equivalent value for A(H3N2) in the USA and Oceania is approximately 1–2 years [32], indicating that Victoria-lineage viruses have slower lineage turnover than A(H3N2) viruses. In contrast to the Northern and Southern temperate regions, the genetic diversity of Victoria-lineage viruses in SC/SEA is more constant, with multiple co-circulating clades in this region (Fig 8C, S10C and S11C Figs). These SC/SEA clades of Victoria-lineage are longer-lasting, with an average TMRCA of 5.1 years (4.7–5.7 years). In contrast, the average TMRCA estimates for Yamagata-lineage viruses are similar at 6.5 (5.9–7.1) in the USA, 7.2 (6.5–7.8) years in Europe, 6.3 (5.7–6.9) in SC/SEA, and 6.7 (6.1–7.3) years in Oceania, highlighting that a similar level of diversity of Yamagata-lineage viruses exists throughout the world due to the co-existence of the two extant Yamagata-lineage clades.

## Discussion

Here we report the global full-genome molecular epidemiology, antigenic evolution, and phylodynamics of influenza B viruses, putting this important human pathogen into a similar context as in analysis of influenza A viruses. Results were obtained from viruses collected between 1987–2015, including the complete genomes of 2,651 unique viruses. Full virus genome analysis show that in contrast to influenza B Victoria-lineage viruses that undergo reassortment between clades, Yamagata-lineage viruses form two persisting co-circulating clades that genetically diverge across the whole virus genome. Yamagata-lineage clade 2 and clade 3 virus populations have a prolonged absence of intra-Yamagata-lineage reassortment, resulting in the long-term maintenance of separate genome constellations. Moreover, estimated timings of this split reveal that the divergence of Yamagata-lineage viruses began much earlier than previously suggested by analysis of HA and NA phylogenies alone. Evolutionary divergence into two distinct genetic clades began with PB1 over twenty years ago, followed by PA and then the remaining genes. Similar observations were made regarding the maintenance of distinct Yamagata- and Victoria-lineages in PB2, PB1, and HA genes, potentially driven by “reassortment incompatibility” [17,33]. This idea has been tested and supported recently by *in vitro* studies [23]. However, unlike the separation between Yamagata- and Victoria-lineage viruses, which is currently restricted to a PB2-PB1-HA complex, the differentiation between the clades of the Yamagata-lineage is maintained across all genes. Interestingly, we observed greater Yamagata/Victoria inter-lineage reassortment for NA and NP than Yamagata intra-lineage reassortment. However, as there are fewer whole-genome sequences than individual HA and NA genes, it is possible that reassortment events between Yamagata-lineage clades remain undetected at low frequencies or in poorly sampled regions of the world.

The co-divergence of the Yamagata-lineage genes relates to experimental studies that suggest that coevolution of PB1 with other influenza genes is important for virus fitness for influenza A viruses [34,35]. Specifically, evidence suggests that optimal PB1-PA interaction is important for efficient polymerase activity and is essential for *in vitro* influenza A virus viability [34]. This is underpinned by an influenza A polymerase model proposing that initial binding between PB1 and PA is necessary for efficient transport to the nucleus and subsequent interaction with PB2 to assemble the polymerase complex [36,37]. PB1 has also been associated with co-selection of virus-matched HA and NA glycoproteins, with reduced virus growth and antigen yield being observed when miss-matched *in vitro* [33,35,38]. Here we observe mutations fixed on the Yamagata-lineage PB1 and PA phylogeny trunk branches at two amino acids (PB1-I357V and PA-I617V) in contact areas of PB1 and PA for Yamagata-lineage clade 2 viruses, one of which was previously functionally characterized [29], and two amino acids (PB1-K652R and PB1-H38Y) associated with PB1/vRNA interaction for Yamagata-lineage clade 3 viruses. The functional significance of these requires testing; however, these data suggest that adaptation of influenza B virus fitness through polymerase activity can occur by at least two mechanisms.

Work here also highlights the importance of model selection for antigenic drift analyses and supports the view that Victoria and Yamagata lineages have comparable rates of antigenic drift [20] in contrast to differences in estimated Influenza B virus antigenic drift rates from previous reports [18]. Detecting selection in influenza viruses is challenging when using traditional statistical tests based on dN/dS ratios, as such ratios are sensitive to recurrent selection at individual sites [39]. Further, adaptations that arise from egg [40,41] and cell-culture [42,43] passaging often appear as recurring mutations, also confounding analyses, whereas analyzing the phylogenetic distribution of mutations can assist in the detection of positive selection. Characterizing amino acid substitutions that occur along the trunk of Yamagata- and Victoria-lineage gene phylogenies, identifies changes that become fixed in the virus population across seasons [44,45], and are thus less likely to be passage artefacts. Notably, we did not detect trunk substitutions at HA residues 196/197 or 198/199, which are known to be highly variable and associated with adaptation to propagation in eggs [40,41].

The HA gene (and encoded glycoprotein) has been the focus of much influenza research, owing to its role in immune escape. A recent study on the global circulation patterns of influenza HA genes noted the persistence of influenza B virus clades, particularly Victoria-lineage clades, which circulated exclusively in China and India for longer periods of time before migrating to other regions [19]. Our whole-genome analysis indicates that geographical constraint extends to other genes of Victoria-lineage viruses, notably with greater levels of genetic diversity for NA, M1, and NS1 detected in SC/SEA compared to other geographic regions. It remains unclear how the spatial structure of Victoria-lineage diversity is maintained or why Yamagata-lineage viruses do not also show this spatial pattern. Based on the incomplete availability of influenza B virus genome sequences, particularly from the Indian subcontinent, the existence of other Yamagata- or Victoria-lineage clades may have gone undetected in our analysis. Further, we cannot exclude the possibility that seemingly geographically-constrained virus populations have gone undetected in other regions, for example in Europe outside of our sampling window. Nevertheless, high levels of intra- and inter-lineage reassortment in the Victoria-lineage are seen and considerably affect genetic diversity, with multiple distinct genotypes generated through reassortment events. In particular, introductions of the SC/SEA Victoria-lineage NA into other geographic regions was associated with reassortant viruses containing the Yamagata-lineage HA and genes (Fig 2). As Yamagata-lineage viruses have been associated with a slightly older age of infection [10,13,18] and associated with more frequent air travel [19], this may contribute to the global migration of these reassortant viruses.

Analysis of Victoria- and Yamagata-lineage viruses shows differences in modes of antigenic evolution. Structural mapping of amino acid changes in HA confirmed the genetic drift estimates, as the accumulation of adaptations in antigenically-relevant sites in each lineage was comparable. The majority of phylogeny trunk substitutions in influenza B HA appear in the globular head and do not map to the stalk region of HA. Whereas Victoria-lineage viruses experience antigenic drift and turnover of antigenically-distinct viruses, the genetic and antigenic bifurcation of Yamagata-lineage viruses has enabled these viruses to alternate between two antigenic types over time. This provides a mechanism for generating antigenic novelty, as previously proposed [46]. This model is supported by the amino acid reconstruction analysis here, as two substitutions at residues located near the RBS (sites 150 and 202) accumulated along the trunk of Yamagata-lineage clade 3, but not in clade 2, potentially affecting antigenicity.

The emergence and co-existence of two major antigenic Yamagata-lineage clades in a region has implications for the epidemiological dynamics of influenza B viruses. For example, Yamagata-lineage viruses dominated influenza B viruses in Malaysia in 2013 after a Victoria-lineage dominated season in 2012–2013. However, in 2014 the Yamagata-lineage continued to dominate in the influenza B virus population, through a shift from clade 2 to clade 3 viruses [13]. This shift in patterns of dominance supports the idea that essentially three ‘lineages’ of influenza B virus co-circulated, with distinct genotypes and antigenicity. Consequently, the persistence of two antigenically-distinct Yamagata-lineage clades may complicate vaccine virus selection. In contrast, we found that Victoria-lineage clade 1a and clade 1b not only genetically reassort, but also occupy the same antigenic dimensions in antigenic map-space, suggesting the WHO-proposed distinction of contemporary Victoria-lineage viruses may not be antigenically relevant. The future coupling of influenza B virus whole genome sequencing and antigenic mapping may well help in global vaccine selection and development of new immunization strategies. The additional whole-genome sequencing data and measurements of antigenic properties of HA presented here, particularly from under-sampled geographic regions, contributes to ongoing public health surveillance of influenza viruses. Our findings provide a better understanding of the interplay of epidemiological, immune-driven, and molecular factors driving the evolution and spread of influenza B viruses worldwide.

## Materials and methods

### Ethics statement

Samples (specimens, clinical samples, or virus isolates) were received by the WHO Collaborating Centre (WHO CC) in London (The Crick Institute, formerly the MRC National Institute for Medical Research) from WHO National Influenza Centers (NICs) and taken with informed consent obtained in each country as laboratories within the WHO Global Influenza Surveillance and Response System (GISRS) for the purposes of global surveillance of influenza under the WHO Global Influenza Program. Samples were anonymized prior to sharing with the WHO CC for influenza B genomic RNA extraction and Institutional Review Board review was not applicable.

### Sample collection

Samples were collected between 2007 and 2013 from 55 countries across Europe, Africa, the Middle East, Asia, and South America. Samples for extraction were chosen based on lack of recovery of virus (clinical specimens) and unusual profiles emerging from HI assays with a panel of post-infection ferret antisera, along with a representative number of viruses showing ‘normal’ HI profiles.

### RT-PCR amplification and sequencing

Amplification was performed using the SuperScriptIII One-Step RT-PCR system with Platinum *Taq* DNA High Fidelity polymerase (Invitrogen) in two reactions. Each reaction contained 25μl Reaction Mix (2x), 17μl DNase/RNase-free water, 1μl of each primer (10μM), 1μl SuperScriptIII RT/Platinum Taq High Fidelity and 5μl of the template RNA. Primers used for the HA, NP, NA, MP, and NS genes were: FluB-S1-F (5’ GCC GGA GCT CTG CAG ATA TCA GCA GAA GCA 3’) and FluB-S1-R (GCC GGA GCT CTG CAG ATA TCA GTA GWA RYA A 3’). Primers used for the polymerase complex genes (PB2, PB1, PA) were: FluB(555)-L1-F (5’ CTG AGT CCG AAC ATT GAG AGC AGA AGC G 3’) and FluB(555)-L1-R (5’ CTG AGT CCG AAC ATT GAG AGT AGA AAC AC 3’) [47]. The cycling conditions were 42°C for 15 min, 55°C for 15 min, 60°C for 5 min, 96°C for 2 min, and then 5 cycles (94°C for 30 s, 45°C for 30 s, slow ramp (0.5°C /sec from 45°C to 66°C) and 68°C for 3 min), followed by 35 cycles (96°C for 30 s, 66°C for 30 s, and 68°C for 3 min) and finally 68°C for 5 min with subsequent examination of amplicons by agarose gel electrophoresis. Amplicons were pooled and sequenced on Illumina MiSeq or HiSeq 2000 platforms using the paired-end 150bp technology. The resultant reads were quality-controlled using QUASR version 7.01 [48] to remove primer sequences, trim low-quality bases from the 3’-ends of reads until the median Phred-scaled quality was 35 and filter reads shorter than 145bp. All raw sequencing reads are available in the European Nucleotide Archive (ENA) under study accessions PRJEB19198 and PRJEB2261.

### Genome assembly

Genomes were generated using *de novo* assembly and reference-based mapping methods. In brief, quality-controlled reads were *de novo* assembled using the SPAdes genome assembler version 2.4.0 [49] with kmer size 127 and minimum contiguous sequence (contig) size of 300. Resulting contigs were arranged by genomic segment and filtered to retain those covering at least 70% of the open reading frame for each segment. In the case where multiple contigs were assembled for a segment, a custom Python script was used to estimate the relative abundance of each contig in the reads (*i*.*e*. to determine composition of variants) and retain the majority variant. For reference-based mapping, unique references were selected for each sample by performing a BLAST search on a subset of the reads and retaining the best match for each segment. Reads were mapped against the reference sequences using SMALT version 0.5.0 [50], and consensus sequences generated using SAMtools version 0.1.8 [51] and QUASR version 7.01 [48]. Sequences generated in this study are available in GISAID under accession numbers listed in S3 Table.

### Sequence collation and alignment

All available influenza B virus gene segment sequences, excluding artificial recombinant and laboratory-generated variants, were downloaded from the NCBI Influenza Virus Resource (IVR) [52] and GISAID (http://gisaid.org) repositories on 28 August 2015. Acknowledgement of the sources of the GISAID sequences is given in S4 Table and accession numbers of GenBank sequences are listed in S5 Table. After duplicate samples and sequences containing less than 70% of the segment coding sequence were removed, the downloaded sequences were combined with the 413 genome sequences generated for this study (representing 382 unique viruses), resulting in a dataset containing 2651 unique, complete genome sequences, (from 2992 PB1, 3090 PB2, 3012 PA, 9167 HA, 3178 NP, 6608 NA, 3403 MP, and 5159 NS sequences) sampled worldwide between 1987 and 2015. Separate alignments were constructed for the longest coding region of each segment (PB2, PB1, PA, HA, NP, NA, M1, NS1) in AliView version 1.17.1 [53]. To reduce sampling bias from over-represented regions in the time-resolved phylogenetic reconstructions, we downsampled epidemiologically-linked isolates while maintaining phylogenetic structure, temporal range and spatial distribution.

### Phylogenetic analysis

Maximum likelihood (ML) phylogenies for each segment were estimated using RAxML version 7.8.6 [54] under a general-time reversible (GTR) nucleotide substitution model with gamma-distributed rates to represent among-site heterogeneity. Clade confidence was estimated by bootstrapping with 1,000 pseudo-replicates. Trees were visualized, rooted to the oldest virus and colour-coded by lineage and clade using FigTree version 1.4.2 (http://tree.bio.ed.ac.uk/software/figtree/). The resulting phylogenetic trees were inspected by linear regression and residual analysis using TempEst v1.4 [55] to identify incorrectly dated or anomalous sequences, which were subsequently removed from the alignments.

### Molecular clock-dating and evolutionary analysis

Molecular clock phylogenies were inferred for each gene segment using the Markov chain Monte Carlo (MCMC) method implemented in BEAST version 1.8.0 [56]. Separate Victoria- and Yamagata-lineage phylogenies were inferred for the PB2, PB1, and HA genes. For all runs, the SRD06 nucleotide substitution model [57] was used, along with a strict molecular clock, as suggested by the linear regression analysis, and a Bayesian Skyride coalescent prior [58]. At least two MCMC chains were run for 200 million states, and combined with a 10% burn-in and sampling every 40,000 states. Mean pairwise diversity measures and 95% highest posterior densities across 9,000 trees were inferred for viruses from each major geographic region in yearly time intervals using PACT (http://bedford.io/projects/PACT/). Amino acid substitutions along the HA phylogenies were inferred using ‘renaissance’ counting ancestral reconstruction methods [59,60]. The ‘trunk’ branches of each phylogenetic tree were defined by tracing from the most recent contemporaneous samples back to the oldest. Nonsynonymous substitutions along the trunk lineage were calculated in year time intervals to determine the mean nonsynonymous substitutions/year count and 95% highest posterior densities across a posterior set of 1000 trees.

### Genotype assignment

Viruses were categorized into major Yamagata- and Victoria-clades, as previously reported in WHO influenza centre reports for HA and NA genes (https://www.crick.ac.uk/research/worldwide-influenza-centre/annual-and-interim-reports), from the ML and time-resolved phylogenies where viruses grouped together in well-supported clades (bootstrap value >60% and/or posterior probability >0.6). Each gene was assigned to one of the defined clades to generate a complete genotype for each sample. Phylogenetics trees were annotated with resulting genotypes and visualized in R using the ggtree package [61]. Data analysis and visualization scripts are available in Github repository https://github.com/pclangat/global-fluB-genomes.

### Antigenic data and integrated cartography

We compiled HI measurements and HA sequence data, which were previously published [20] or collected by the WHO Collaborating Centre (WHO CC) in London. Known egg-adapted viruses were removed, resulting in a final HI dataset of 309 Victoria- and 308 Yamagata-lineage viruses isolated from 1988 to 2013. We implemented a Bayesian multidimensional scaling (BMDS) cartographic model to jointly infer antigenic and phylogenetic relationships of the viruses as previously described [20,62]. Briefly, MCMC was used to sample virus and serum locations in two antigenic dimensions, as well as virus avidities, serum potencies, MDS precision, and virus and serum location precisions, using an empirical tree distribution of 1,000 posterior trees inferred for HA sequences separately (as detailed above). Two antigenic dimensions were specified based on previous findings that two-dimensional models provide the best predictive power for antigenic mapping of influenza B virus [20]. MCMC chains were run for 500 million states with sampling every 200,000 states with a 10% burn-in, and checked for convergence in Tracer v1.6 (http://tree.bio.ed.ac.uk/software/tracer/). We obtained a total of 2,000 trees from which the maximum clade credibility tree was summarized in TreeAnnotator v1.8.2. We estimated the rate of antigenic drift for each lineage, by calculating the mean Euclidean distance in antigenic units (AU) of all antigenic map locations at yearly time intervals from the inferred phylogenetic root. From this time series of Euclidean distances, we estimated the rates of antigenic drift (in AU/year) using linear regression. 95% highest posterior density (HPD) estimates were used to measure the statistical uncertainty in these drift rate inferences from the posterior sample of trees. Source data, including BEAST input XML files, HI tables, and output trees are available in Dryad repository https://doi:10.5061/dryad.s1d37 [64].

### Structural mapping

Amino acid substitutions occurring along the trunk of each lineage were visualized on the crystal structures of the HA trimers for viruses of the Yamagata-lineage B/Yamanashi/166/98 (PDB ID: 4M40, [63]) and Victoria-lineage B/Brisbane/60/2008 (PDB ID: 4FQM, [27]), and influenza B virus PB2-PB1-PA polymerase complex bound to viral RNA (PDB ID: 5MSG, [30]) using PyMOL Molecular Graphics System, Version 1.7.6.0, Schrödinger, LLC. Structural features were mapped as described in S8 Fig.

## Supporting information

S1 Fig Maximum-clade credibility trees for all major influenza B virus genes. Branches of phylogenies are labeled with amino acid substitutions occurring along the phylogenetic ‘trunk’ and are colored by well-supported clade distinctions. Nodes with greater than 0.70 posterior probability support are shown with circle node shapes.
(PDF)

S2 Fig MCC tree inferred from 522 Yamagata-lineage PB1 gene sequences and corresponding genotype constellations. See Fig 2 legend for details.
(PDF)

S3 Fig MCC tree inferred from 902 Victoria-lineage NA gene sequences and corresponding genotype constellations. See Fig 2 legend for details.
(PDF)

S4 Fig Antigenic and genetic evolutionary relationships of influenza B viruses inferred using a BMDS model with fixed serum potencies and virus avidities. See Fig 4 legend for further details.
(PDF)

S5 Fig Antigenic map configurations for 309 Victoria-lineage and 308 Yamagata-lineage viruses inferred under BMDS models with co-estimated (A, B) and fixed (C,D) serum potencies and virus avidities, shown in two antigenic dimensions and one time dimension. See Fig 4 legend for further details and S1 and S2 Videos for animated visualizations.
(PDF)

S6 Fig Estimated global relative frequencies for Yamagata-lineage clade 2 and clade 3 viruses. As reported on nextflu.org (accessed 8 August 2016).
(PDF)

S7 Fig Structural mapping of phylogenetic ‘trunk’ amino acid substitutions on Victoria-lineage and Yamagata-lineage HA trimers. HA1 units are in darker grey while HA2 are white-grey, with the RBS shown in pink. (A) Victoria-lineage virus B/Brisbane/60/2008 (PDB: 4FQM), (B) Yamagata-lineage virus B/ Yamanashi/166/98 (PDB: 4M40) and (C) rotated Yamagata-lineage virus B/Yamanashi/166/98. On the front- facing selected HA1 monomer (darkest grey), trunk mutations are labeled with residue number and coloured by location: in antigenic epitope (orange) or other (blue), while they are shown in black on the other two monomers for simplicity.
(PDF)

S8 Fig Structure-based sequence alignment of influenza B/Yamagata and B/Victoria HA1. Alignment was performed with Multalin (Corpet 1988) and plotted with ESPRIPT (Gouet et al. 1999). Secondary structure elements were assigned using the crystal structure of hemagglutinin influenza virus B/ Yamanashi/166/1998 in complex with avian-like receptor LSTa (PDB accession number 4M44) (Ni et al. 2013). Secondary structure elements are shown with an arrow and helices are shown as spirals. Residues which are highlighted red are fully conserved, residues which are colored red are partially conserved, and residues which are black are not conserved. Residues which are solvent accessible (as determined by ESPRIPT) are highlighted by black (fully exposed) and gray (partially exposed) bars below the sequence. Residues located at the receptor binding site were determined using the PISA EBI server (Krissinel and Henrick 2007) and are annotated with pink bars below the sequence. An asterisk is placed at positions at sites which do not map on or nearby the major epitopes. Four previously described (Wang et al. 2008) major epitopes on the Influenza B virus are annotated below the sequence with orange bars. Residues in close- proximity to these regions which undergo frequent amino-acid substitutions in influenza B virus HA (Ni et al. 2013; Wang et al. 2008; Nunes et al. 2008; Pechirra et al. 2005; Shen et al. 2009) are annotated with green bars.
(PDF)

S9 Fig Structural mapping of additional Yamagata-lineage ‘clade-defining’ trunk substitutions on influenza B polymerase complex. Substitutions not located at potential PB1/PA or PB1/vRNA interface regions are highlighted as spheres coloured by emergence in Yamagata-lineage clade 2 (orange) or clade 3 (red) viruses. See Fig 6 legend for details.
(PDF)

S10 Fig Time-resolved HA gene phylogenies of influenza B viruses isolated in four major global regions from 2001–2014. Clades are highlighted in colored blocks: Yamagata-lineage B/Florida/4/2006 (FR06) clade shown in yellow. See Fig 8 legend for other details.
(PDF)

S11 Fig Relative genetic diversity of HA genes of influenza B viruses circulating in different regions of the world. Effective population sizes over time inferred by Bayesian skyride analysis for HA of Victoria- (blue) and Yamagata-lineage (red) viruses isolated in (A) the USA, (B) Europe, (C) SC/SEA and (D) Oceania. Solid lines represent median values and shaded areas represent 95% highest probability densities (HPD) credible intervals across MCMC samples.
(PDF)

S12 Fig Genetic diversity of gene segments over time in less-sampled geographic regions. Time series of mean pairwise diversity for viruses collected from countries of the African Region of WHO (AFRO) and Eastern Mediterranean Region of WHO (EMRO) as listed on http://www.who.int/influenza/gisrs_laboratory/national_influenza_centres/list/en/ (Accessed 8 August 2016). Due to limited sampling, these regions are not discussed in the main manuscript. See Fig 6 legend for further details.
(PDF)

S13 Fig Time-resolved HA and NA gene phylogenies of influenza B viruses isolated in WHO AFRO and EMRO regions from 2001–2014. Due to limited sampling, these regions are not discussed in the main text. See Fig 5 legend for further details.
(PDF)

S1 Table Inferred trunk mutations and dates of emergence for influenza B virus gene phylogenies. (XLS)

S2 Table Structural features and nature of select amino acid substitutions inferred along trunk lineages of Victoria and Yamagata HA phylogenies. (XLS)

S3 Table GISAID accessions and meta data for sequences generated in this study. (XLS)

S4 Table GISAID acknowledgement table for sequences used in this study. (XLS)

S5 Table NCBI accessions and meta data for sequences used in this study. (XLS)

S1 Video Animated rotating view of three-dimensional antigenic map visualization for Victoria-lineage viruses shown in S5A Fig. (MP4)

S2 Video Animated rotating view of three-dimensional antigenic map visualization for Yamagata-lineage viruses shown in S5B Fig. (MP4)

## References

[1] McCullers JA, Hayden FG. Fatal influenza B infections: time to reexamine influenza research priorities. J Infect Dis. 2012 3 15;205(6):870–2. doi: 10.1093/infdis/jir865 22291194 10.1093/infdis/jir865

[2] Yang J-R, Huang Y-P, Chang F-Y, Hsu L-C, Lin Y-C, Huang H-Y, et al Phylogenetic and evolutionary history of influenza B viruses, which caused a large epidemic in 2011–2012, Taiwan. PLoS One. 2012 10 12;7(10):e47179 doi: 10.1371/journal.pone.0047179 23071751 10.1371/journal.pone.0047179 PMC3470568

[3] Garg S, Moore Z, Lee N, McKenna J, Bishop A, Fleischauer A, et al A cluster of patients infected with I221V influenza b virus variants with reduced oseltamivir susceptibility—North Carolina and South Carolina, 2010–2011. J Infect Dis. 2013 3 15;207(6):966–73. doi: 10.1093/infdis/jis776 23242536 10.1093/infdis/jis776

[4] Tan Y, Guan W, Lam TT-Y, Pan S, Wu S, Zhan Y, et al Differing epidemiological dynamics of influenza B virus lineages in Guangzhou, southern China, 2009–2010. J Virol. 2013 11;87(22):12447–56. doi: 10.1128/JVI.01039-13 24027322 10.1128/JVI.01039-13 PMC3807886

[5] Koutsakos M, Nguyen THO, Barclay WS, Kedzierska K. Knowns and unknowns of influenza B viruses. Future Microbiol. 2016;11(1):119–35. doi: 10.2217/fmb.15.120 26684590 10.2217/fmb.15.120

[6] Kanegae Y, Sugita S, Endo A, Ishida M, Senya S, Osako K, et al Evolutionary pattern of the hemagglutinin gene of influenza B viruses isolated in Japan: cocirculating lineages in the same epidemic season. J Virol. 1990 6;64(6):2860–5. 2335820 10.1128/jvi.64.6.2860-2865.1990 PMC249468

[7] Rota PA, Wallis TR, Harmon MW, Rota JS, Kendal AP, Nerome K. Cocirculation of two distinct evolutionary lineages of influenza type B virus since 1983. Virology. 1990 3;175(1):59–68. 2309452 10.1016/0042-6822(90)90186-u

[8] Lo Y-C, Chuang J-H, Kuo H-W, Huang W-T, Hsu Y-F, Liu M-T, et al Surveillance and vaccine effectiveness of an influenza epidemic predominated by vaccine-mismatched influenza B/Yamagata-lineage viruses in Taiwan, 2011–12 season. PLoS One. 2013 3 5;8(3):e58222 doi: 10.1371/journal.pone.0058222 23472161 10.1371/journal.pone.0058222 PMC3589334

[9] Reed C, Meltzer MI, Finelli L, Fiore A. Public health impact of including two lineages of influenza B in a quadrivalent seasonal influenza vaccine. Vaccine. 2012 3 2;30(11):1993–8. doi: 10.1016/j.vaccine.2011.12.098 22226861 10.1016/j.vaccine.2011.12.098

[10] Tan Y, Guan W, Lam TT-Y, Pan S, Wu S, Zhan Y, et al Differing epidemiological dynamics of influenza B virus lineages in Guangzhou, southern China, 2009–2010. J Virol. 2013 11;87(22):12447–56. doi: 10.1128/JVI.01039-13 24027322 10.1128/JVI.01039-13 PMC3807886

[11] Nakagawa N, Higashi N, Nakagawa T. Cocirculation of antigenic variants and the vaccine-type virus during the 2004–2005 influenza B virus epidemics in Japan. J Clin Microbiol. 2009 2;47(2):352–7. doi: 10.1128/JCM.01357-08 19091818 10.1128/JCM.01357-08 PMC2643680

[12] Roy T, Agrawal AS, Mukherjee A, Mishra AC, Chadha MS, Kaur H, et al Surveillance and molecular characterization of human influenza B viruses during 2006–2010 revealed co-circulation of Yamagata-like and Victoria-like strains in eastern India. Infect Genet Evol. 2011 10;11(7):1595–601. doi: 10.1016/j.meegid.2011.05.022 21708292 10.1016/j.meegid.2011.05.022

[13] Oong XY, Ng KT, Lam TT-Y, Pang YK, Chan KG, Hanafi NS, et al Epidemiological and Evolutionary Dynamics of Influenza B Viruses in Malaysia, 2012–2014. PLoS One. 2015 8 27;10(8):e0136254 doi: 10.1371/journal.pone.0136254 26313754 10.1371/journal.pone.0136254 PMC4552379

[14] Sam I-C, I-Ching S, Su YCF, Chan YF, Nor’E SS, Ardalinah H, et al Evolution of Influenza B Virus in Kuala Lumpur, Malaysia, between 1995 and 2008. J Virol. 2015;89(18):9689–92. doi: 10.1128/JVI.00708-15 26136576 10.1128/JVI.00708-15 PMC4542393

[15] Tewawong N, Suwannakarn K, Prachayangprecha S, Korkong S, Vichiwattana P, Vongpunsawad S, et al Molecular epidemiology and phylogenetic analyses of influenza B virus in Thailand during 2010 to 2014. PLoS One. 2015 1 20;10(1):e0116302 doi: 10.1371/journal.pone.0116302 25602617 10.1371/journal.pone.0116302 PMC4300180

[16] Chen R, Rubing C, Holmes EC. The Evolutionary Dynamics of Human Influenza B Virus. J Mol Evol. 2008;66(6):655–63. doi: 10.1007/s00239-008-9119-z 18504518 10.1007/s00239-008-9119-z PMC3326418

[17] Dudas G, Bedford T, Lycett S, Rambaut A. Reassortment between Influenza B Lineages and the Emergence of a Coadapted PB1–PB2–HA Gene Complex. Mol Biol Evol. 2015 1 1;32(1):162–72. doi: 10.1093/molbev/msu287 25323575 10.1093/molbev/msu287 PMC4271528

[18] Vijaykrishna D, Holmes EC, Joseph U, Fourment M, Su YCF, Halpin R, et al The contrasting phylodynamics of human influenza B viruses. Elife. 2015 1 16;4:e05055 doi: 10.7554/eLife.05055 25594904 10.7554/eLife.05055 PMC4383373

[19] Bedford T, Steven R, Barr IG, Shobha B, Mandeep C, Cox NJ, et al Global circulation patterns of seasonal influenza viruses vary with antigenic drift. Nature. 2015;523(7559):217–20. doi: 10.1038/nature14460 26053121 10.1038/nature14460 PMC4499780

[20] Bedford T, Suchard MA, Lemey P, Dudas G, Gregory V, Hay AJ, et al Integrating influenza antigenic dynamics with molecular evolution. Elife. 2014 2 4;3:e01914 doi: 10.7554/eLife.01914 24497547 10.7554/eLife.01914 PMC3909918

[21] McCullers JA, Wang GC, He S, Webster RG. Reassortment and insertion-deletion are strategies for the evolution of influenza B viruses in nature. J Virol. 1999 9;73(9):7343–8. 10438823 10.1128/jvi.73.9.7343-7348.1999 PMC104260

[22] Nerome R, Hiromoto Y, Sugita S, Tanabe N, Ishida M, Matsumoto M, et al Evolutionary characteristics of influenza B virus since its first isolation in 1940: dynamic circulation of deletion and insertion mechanism. Arch Virol. 1998;143(8):1569–83. 9739335 10.1007/s007050050399

[23] Kim JI, Lee I, Park S, Bae J-Y, Yoo K, Lemey P, et al Reassortment compatibility between PB1, PB2, and HA genes of the two influenza B virus lineages in mammalian cells. Sci Rep. 2016 6 8;6:27480 doi: 10.1038/srep27480 27270757 10.1038/srep27480 PMC4897687

[24] Barr IG, McCauley J, Cox N, Daniels R, Engelhardt OG, Fukuda K, et al Epidemiological, antigenic and genetic characteristics of seasonal influenza A(H1N1), A(H3N2) and B influenza viruses: basis for the WHO recommendation on the composition of influenza vaccines for use in the 2009–2010 northern hemisphere season. Vaccine. 2010 2 3;28(5):1156–67. doi: 10.1016/j.vaccine.2009.11.043 20004635 10.1016/j.vaccine.2009.11.043

[25] Barr IG, Russell C, Besselaar TG, Cox NJ, Daniels RS, Donis R, et al WHO recommendations for the viruses used in the 2013–2014 Northern Hemisphere influenza vaccine: Epidemiology, antigenic and genetic characteristics of influenza A(H1N1)pdm09, A(H3N2) and B influenza viruses collected from October 2012 to January 2013. Vaccine. 2014 8 20;32(37):4713–25. doi: 10.1016/j.vaccine.2014.02.014 24582632 10.1016/j.vaccine.2014.02.014

[26] Wang Q, Cheng F, Lu M, Tian X, Ma J. Crystal Structure of Unliganded Influenza B Virus Hemagglutinin. J Virol. 2008;82(6):3011–20. doi: 10.1128/JVI.02477-07 18184701 10.1128/JVI.02477-07 PMC2259021

[27] Dreyfus C, Laursen NS, Kwaks T, Zuijdgeest D, Khayat R, Ekiert DC, et al Highly conserved protective epitopes on influenza B viruses. Science. 2012 9 14;337(6100):1343–8. doi: 10.1126/science.1222908 22878502 10.1126/science.1222908 PMC3538841

[28] Koel BF, Burke DF, Bestebroer TM, van der Vliet S, Zondag GCM, Vervaet G, et al Substitutions near the receptor binding site determine major antigenic change during influenza virus evolution. Science. 2013 11 22;342(6161):976–9. doi: 10.1126/science.1244730 24264991 10.1126/science.1244730

[29] Wunderlich K, Mayer D, Ranadheera C, Holler A-S, Mänz B, Martin A, et al Identification of a PA-binding peptide with inhibitory activity against influenza A and B virus replication. PLoS One. 2009 10 20;4(10):e7517 doi: 10.1371/journal.pone.0007517 19841738 10.1371/journal.pone.0007517 PMC2759517

[30] Reich S, Guilligay D, Cusack S. An in vitro fluorescence based study of initiation of RNA synthesis by influenza B polymerase. Nucleic Acids Res. 2017 4 7;45(6):3353–68. doi: 10.1093/nar/gkx043 28126917 10.1093/nar/gkx043 PMC5399792

[31] Kuo S-M, Shu-Ming K, Guang-Wu C, Velu AB, Srinivas D, Yi-Ju H, et al Circulating pattern and genomic characteristics of influenza B viruses in Taiwan from 2003 to 2014. J Formos Med Assoc. 2016;115(7):510–22. doi: 10.1016/j.jfma.2016.01.017 27038555 10.1016/j.jfma.2016.01.017

[32] Rambaut A, Pybus OG, Nelson MI, Viboud C, Taubenberger JK, Holmes EC. The genomic and epidemiological dynamics of human influenza A virus. Nature. 2008 5 29;453(7195):615–9. doi: 10.1038/nature06945 18418375 10.1038/nature06945 PMC2441973

[33] Cobbin JCA, Verity EE, Gilbertson BP, Rockman SP, Brown LE. The source of the PB1 gene in influenza vaccine reassortants selectively alters the hemagglutinin content of the resulting seed virus. J Virol. 2013 5;87(10):5577–85. doi: 10.1128/JVI.02856-12 23468502 10.1128/JVI.02856-12 PMC3648160

[34] Perez DR, Donis RO. Functional Analysis of PA Binding by Influenza A Virus PB1: Effects on Polymerase Activity and Viral Infectivity. J Virol. 2001;75(17):8127–36. doi: 10.1128/JVI.75.17.8127-8136.2001 11483758 10.1128/JVI.75.17.8127-8136.2001 PMC115057

[35] Gíria M, Santos L, Louro J, Rebelo de Andrade H. Reverse genetics vaccine seeds for influenza: Proof of concept in the source of PB1 as a determinant factor in virus growth and antigen yield. Virology. 2016 5 27;496:21–7. doi: 10.1016/j.virol.2016.05.015 27240145 10.1016/j.virol.2016.05.015

[36] Fodor E, Smith M. The PA subunit is required for efficient nuclear accumulation of the PB1 subunit of the influenza A virus RNA polymerase complex. J Virol. 2004 9;78(17):9144–53. doi: 10.1128/JVI.78.17.9144-9153.2004 15308710 10.1128/JVI.78.17.9144-9153.2004 PMC506948

[37] Deng T, Sharps J, Fodor E, Brownlee GG. In vitro assembly of PB2 with a PB1-PA dimer supports a new model of assembly of influenza A virus polymerase subunits into a functional trimeric complex. J Virol. 2005 7;79(13):8669–74. doi: 10.1128/JVI.79.13.8669-8674.2005 15956611 10.1128/JVI.79.13.8669-8674.2005 PMC1143706

[38] Cobbin JCA, Ong C, Verity E, Gilbertson BP, Rockman SP, Brown LE. Influenza virus PB1 and neuraminidase gene segments can cosegregate during vaccine reassortment driven by interactions in the PB1 coding region. J Virol. 2014 8;88(16):8971–80. doi: 10.1128/JVI.01022-14 24872588 10.1128/JVI.01022-14 PMC4136297

[39] Bhatt S, Holmes EC, Pybus OG. The genomic rate of molecular adaptation of the human influenza A virus. Mol Biol Evol. 2011 9;28(9):2443–51. doi: 10.1093/molbev/msr044 21415025 10.1093/molbev/msr044 PMC3163432

[40] Robertson JS, Bootman JS, Daniels R, Newman B, Webster RG, Schild GC. Changes in the haemagglutinin op influenza virus during egg adaptation. Virus Res. 1985;3:79. 10.1016/0042-6822(87)90040-7 3629978

[41] Schild GC, Oxford JS, de Jong JC, Webster RG. Evidence for host-cell selection of influenza virus antigenic variants. Nature. 1983;303(5919):706–9. 6190093 10.1038/303706a0

[42] McWhite C, Claire M, Austin M, Wilke CO. Serial passaging causes extensive positive selection in seasonal influenza A hemagglutinin. bioRxiv [Internet]. 2016; http://dx.doi.org/10.1101/038364

[43] Gatherer D. Passage in egg culture is a major cause of apparent positive selection in influenza B hemagglutinin. J Med Virol. 2010 1;82(1):123–7. doi: 10.1002/jmv.21648 19950248 10.1002/jmv.21648

[44] Wolf YI, Viboud C, Holmes EC, Koonin EV, Lipman DJ. Long intervals of stasis punctuated by bursts of positive selection in the seasonal evolution of influenza A virus. Biol Direct. 2006 10 26;1:34 doi: 10.1186/1745-6150-1-34 17067369 10.1186/1745-6150-1-34 PMC1647279

[45] Cobey S, Koelle K. Capturing escape in infectious disease dynamics. Trends Ecol Evol. 2008 10;23(10):572–7. doi: 10.1016/j.tree.2008.06.008 18715671 10.1016/j.tree.2008.06.008

[46] Bedford T, Sarah C, Mercedes P. Strength and tempo of selection revealed in viral gene genealogies. BMC Evol Biol. 2011;11(1):220. 21787390 10.1186/1471-2148-11-220 PMC3199772

[47] Gall A, Hoffmann B, Harder T, Grund C, Beer M. Universal Primer Set for Amplification and Sequencing of HA0 Cleavage Sites of All Influenza A Viruses. J Clin Microbiol. 2008;46(8):2561–7. doi: 10.1128/JCM.00466-08 18562585 10.1128/JCM.00466-08 PMC2519470

[48] Watson SJ, Welkers MRA, Depledge DP, Coulter E, Breuer JM, de Jong MD, et al Viral population analysis and minority-variant detection using short read next-generation sequencing. Philos Trans R Soc Lond B Biol Sci. 2013 3 19;368(1614):. 10.1098/rstb.2012.0205 PMC3678329 23382427

[49] Bankevich A, Nurk S, Antipov D, Gurevich AA, Dvorkin M, Kulikov AS, et al SPAdes: a new genome assembly algorithm and its applications to single-cell sequencing. J Comput Biol. 2012 5;19(5):455–77. doi: 10.1089/cmb.2012.0021 22506599 10.1089/cmb.2012.0021 PMC3342519

[50] Ning Z, Cox AJ, Mullikin JC. SSAHA: a fast search method for large DNA databases. Genome Res. 2001 10;11(10):1725–9. doi: 10.1101/gr.194201 11591649 10.1101/gr.194201 PMC311141

[51] Li H, Handsaker B, Wysoker A, Fennell T, Ruan J, Homer N, et al The Sequence Alignment/Map format and SAMtools. Bioinformatics. 2009 8 15;25(16):2078–9. doi: 10.1093/bioinformatics/btp352 19505943 10.1093/bioinformatics/btp352 PMC2723002

[52] Bao Y, Bolotov P, Dernovoy D, Kiryutin B, Zaslavsky L, Tatusova T, et al The influenza virus resource at the National Center for Biotechnology Information. J Virol. 2008 Jan;82(2):596–601. doi: 10.1128/JVI.02005-07 17942553 10.1128/JVI.02005-07 PMC2224563

[53] Larsson A. AliView: a fast and lightweight alignment viewer and editor for large datasets. Bioinformatics. 2014 11 15;30(22):3276–8. doi: 10.1093/bioinformatics/btu531 25095880 10.1093/bioinformatics/btu531 PMC4221126

[54] Stamatakis A. RAxML-VI-HPC: maximum likelihood-based phylogenetic analyses with thousands of taxa and mixed models. Bioinformatics. 2006 11 1;22(21):2688–90. doi: 10.1093/bioinformatics/btl446 16928733 10.1093/bioinformatics/btl446

[55] Rambaut A, Lam TT, Max Carvalho L, Pybus OG. Exploring the temporal structure of heterochronous sequences using TempEst (formerly Path-O-Gen). Virus Evol. 2016 1;2(1):vew007 doi: 10.1093/ve/vew007 27774300 10.1093/ve/vew007 PMC4989882

[56] Drummond AJ, Rambaut A. BEAST: Bayesian evolutionary analysis by sampling trees. BMC Evol Biol. 2007 11 8;7:214 doi: 10.1186/1471-2148-7-214 17996036 10.1186/1471-2148-7-214 PMC2247476

[57] Shapiro B, Rambaut A, Drummond AJ. Choosing appropriate substitution models for the phylogenetic analysis of protein-coding sequences. Mol Biol Evol. 2006 1;23(1):7–9. doi: 10.1093/molbev/msj021 16177232 10.1093/molbev/msj021

[58] Minin VN, Bloomquist EW, Suchard MA. Smooth skyride through a rough skyline: Bayesian coalescent-based inference of population dynamics. Mol Biol Evol. 2008 7;25(7):1459–71. doi: 10.1093/molbev/msn090 18408232 10.1093/molbev/msn090 PMC3302198

[59] O’Brien JD, Minin VN, Suchard MA. Learning to count: robust estimates for labeled distances between molecular sequences. Mol Biol Evol. 2009 4;26(4):801–14. doi: 10.1093/molbev/msp003 19131426 10.1093/molbev/msp003 PMC2734148

[60] Lemey P, Minin VN, Bielejec F, Kosakovsky Pond SL, Suchard MA. A counting renaissance: combining stochastic mapping and empirical Bayes to quickly detect amino acid sites under positive selection. Bioinformatics. 2012;28(24):3248–56. doi: 10.1093/bioinformatics/bts580 23064000 10.1093/bioinformatics/bts580 PMC3579240

[61] Yu G, Smith DK, Zhu H, Guan Y, Lam TT-Y. ggtree: an r package for visualization and annotation of phylogenetic trees with their covariates and other associated data. Methods Ecol Evol. 2016;8(1):28–36.

[62] Lewis NS, Russell CA, Langat P, Anderson TK, Berger K, Bielejec F, et al The global antigenic diversity of swine influenza A viruses. Elife [Internet]. 2016 4 15;5 Available from: http://dx.doi.org/10.7554/eLife.12217 10.7554/eLife.12217 PMC4846380 27113719

[63] Ni F, Kondrashkina E, Wang Q. Structural basis for the divergent evolution of influenza B virus hemagglutinin. Virology. 2013 11;446(1–2):112–22. doi: 10.1016/j.virol.2013.07.035 24074573 10.1016/j.virol.2013.07.035 PMC3902124

[64] Langat P, Raghwani J, Dudas G, Bowden T, Edwards S, Gall A, Bedford T, Rambaut A, Daniels R, Russell C, Pybus O, McCauley J, Kellam P, Watson S. Data from: Genome-wide evolutionary dynamics of influenza B viruses on a global scale. Dryad Digital Repository. https://doi:10.5061/dryad.s1d37 10.1371/journal.ppat.1006749 PMC5790164 29284042

