# Supplementary material for: Genome-wide evolutionary dynamics of influenza B viruses on a global scale
Source: PLoS Pathog. 2017 Dec 28;13(12):e1006749. doi: 10.1371/journal.ppat.1006749 (PMC5790164; doi:10.1371/journal.ppat.1006749)
Supplement: S2 Fig — See Fig 2 legend for details. (PDF) [file ppat.1006749.s002.pdf]

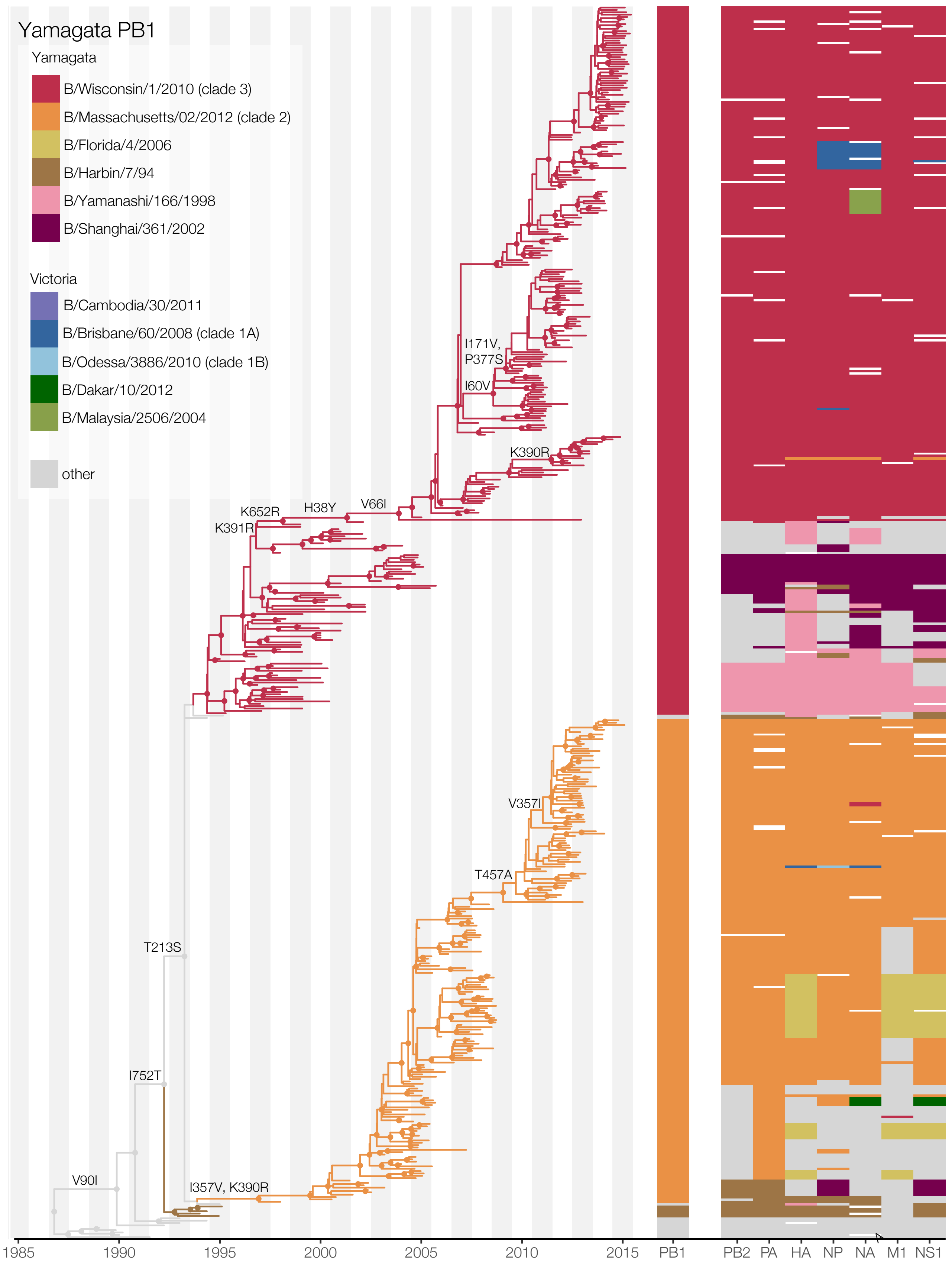

S2 Fig. MCC tree inferred from 522 Yamagata-lineage PB1 gene sequences and corresponding genotype constellations. See Fig 2 legend for details.
