# Supplementary material for: Genome-wide evolutionary dynamics of influenza B viruses on a global scale
Source: PLoS Pathog. 2017 Dec 28;13(12):e1006749. doi: 10.1371/journal.ppat.1006749 (PMC5790164; doi:10.1371/journal.ppat.1006749)
Supplement: S4 Fig — See Fig 4 legend for further details. (PDF) [file ppat.1006749.s004.pdf]

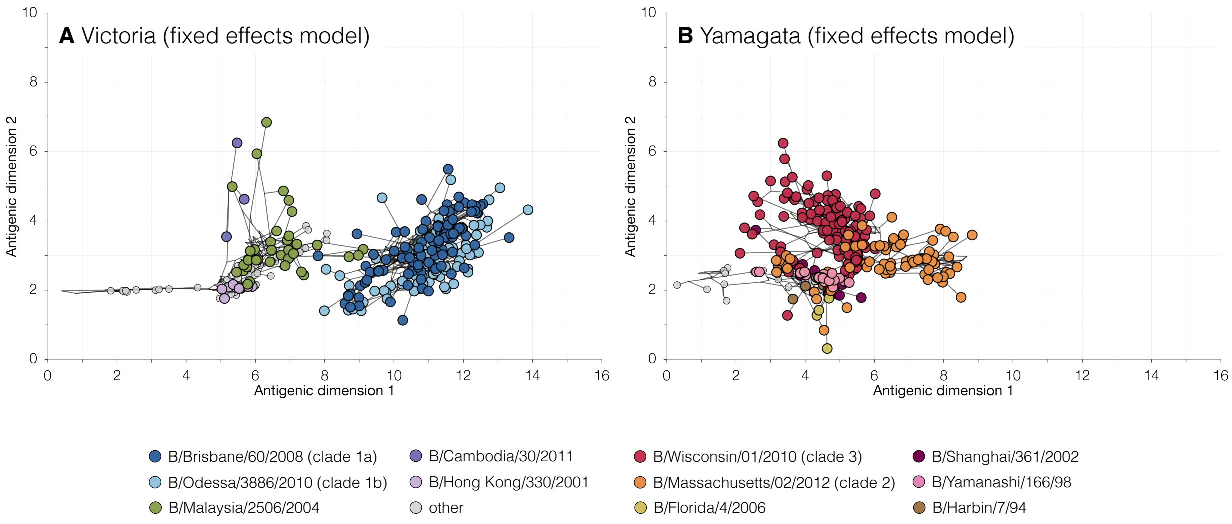

S4 Fig. Antigenic and genetic evolutionary relationships of (A) Victoria-lineage and (B) Yamagata-lineage influenza B viruses inferred using a BMDS model with fixed serum potencies and virus avidities. See Fig 4 legend for further details.
