# Supplementary material for: Genome-wide evolutionary dynamics of influenza B viruses on a global scale
Source: PLoS Pathog. 2017 Dec 28;13(12):e1006749. doi: 10.1371/journal.ppat.1006749 (PMC5790164; doi:10.1371/journal.ppat.1006749)
Supplement: S5 Fig — See Fig 4 legend for further details and S1 and S2 Videos for animated visualizations. (PDF) [file ppat.1006749.s005.pdf]

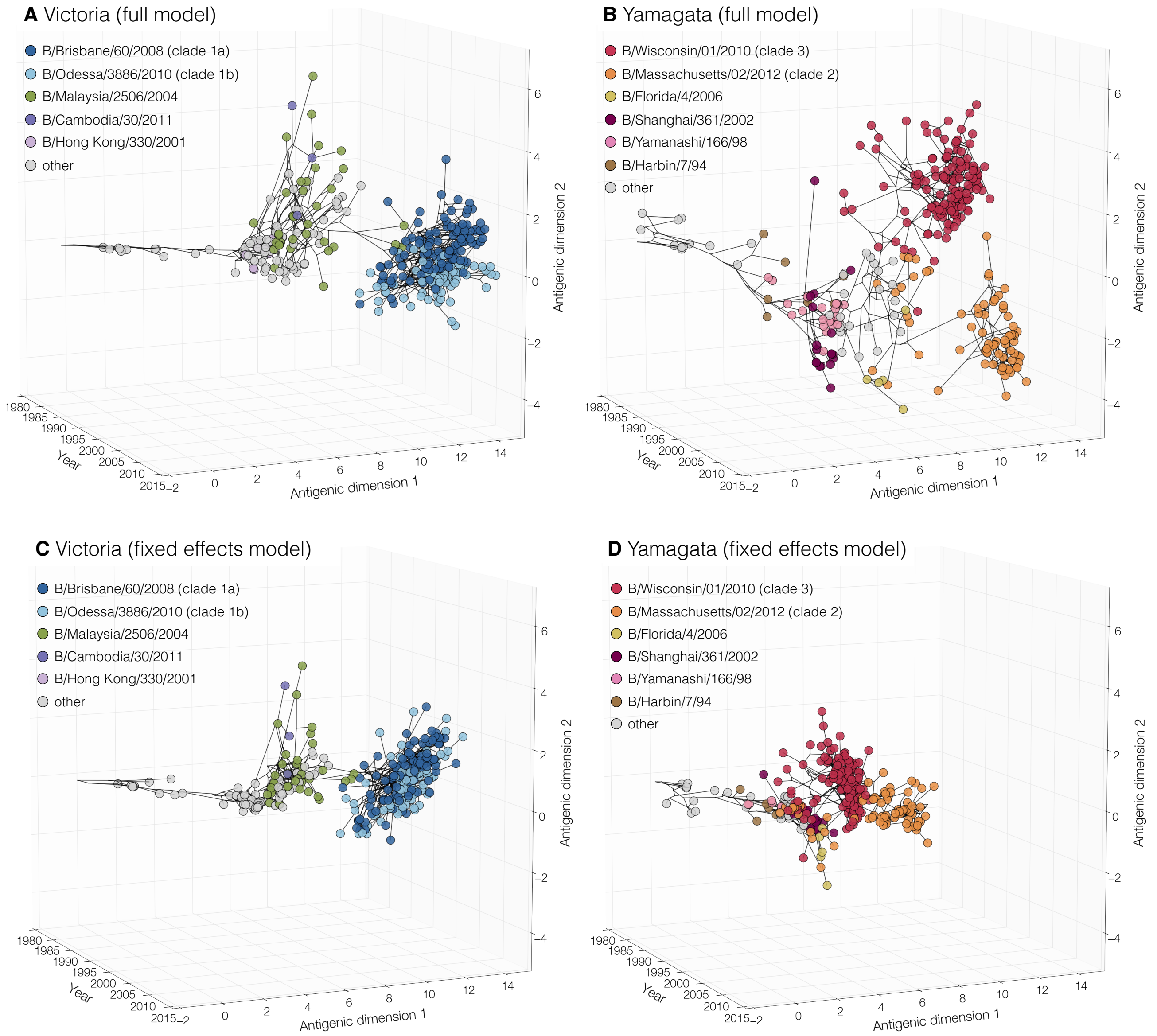

S5 Fig. Antigenic map configurations for 309 Victoria-lineage and 308 Yamagata-lineage viruses inferred under BMDS models with co-estimated (A, B) and fixed (C,D) serum potencies and virus avidities, shown in two antigenic dimensions and one time dimension. See Fig 4 legend for further details and S1 and S2 Videos for animated visualizations.
