# Supplementary material for: Genome-wide evolutionary dynamics of influenza B viruses on a global scale
Source: PLoS Pathog. 2017 Dec 28;13(12):e1006749. doi: 10.1371/journal.ppat.1006749 (PMC5790164; doi:10.1371/journal.ppat.1006749)
Supplement: S6 Fig — As reported on nextflu.org (accessed 8 August 2016). (PDF) [file ppat.1006749.s006.pdf]

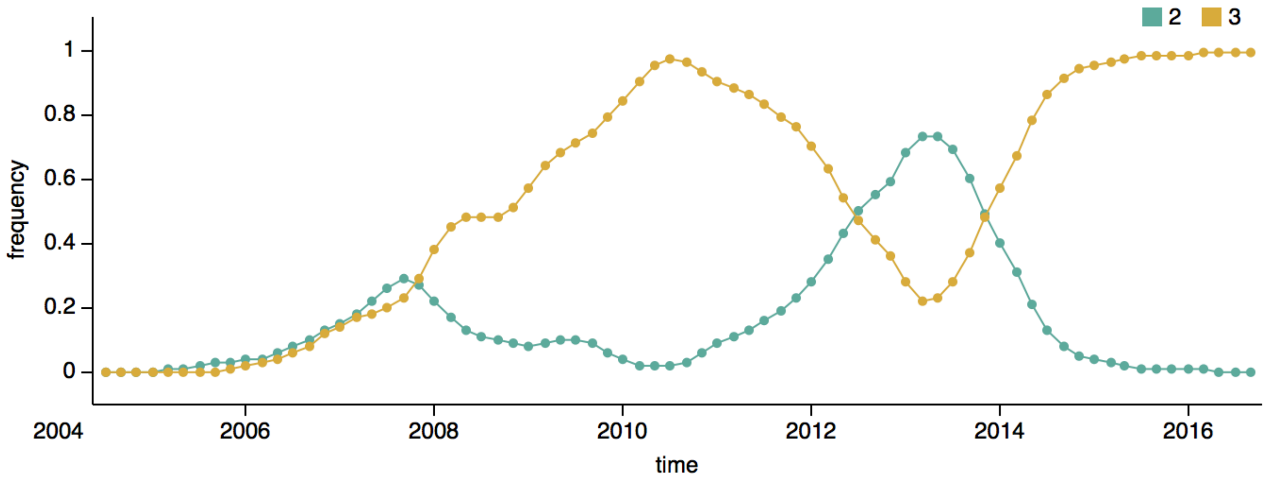

S6 Fig. Estimated global relative frequencies for Yamagata-lineage clade 2 and clade 3 viruses. As reported on nextflu.org (accessed 8 August 2016).
