# Supplementary material for: Genome-wide evolutionary dynamics of influenza B viruses on a global scale
Source: PLoS Pathog. 2017 Dec 28;13(12):e1006749. doi: 10.1371/journal.ppat.1006749 (PMC5790164; doi:10.1371/journal.ppat.1006749)
Supplement: S7 Fig — HA1 units are in darker grey while HA2 are white-grey, with the RBS shown in pink. (A) Victoria-lineage virus B/Brisbane/60/2008 (PDB: 4FQM), (B) Yamagata-lineage virus B/ Yamanashi/166/98 (PDB: 4M40) and (C) rotated Yamagata-lineage virus B/Yamanashi/166/98. On the front- facing selected HA1 monomer (darkest grey), trunk mutations are labeled with residue number and coloured by location: in antigenic epitope (orange) or other (blue), while they are shown in black on the other two monomers for simplicity. (PDF) [file ppat.1006749.s007.pdf]

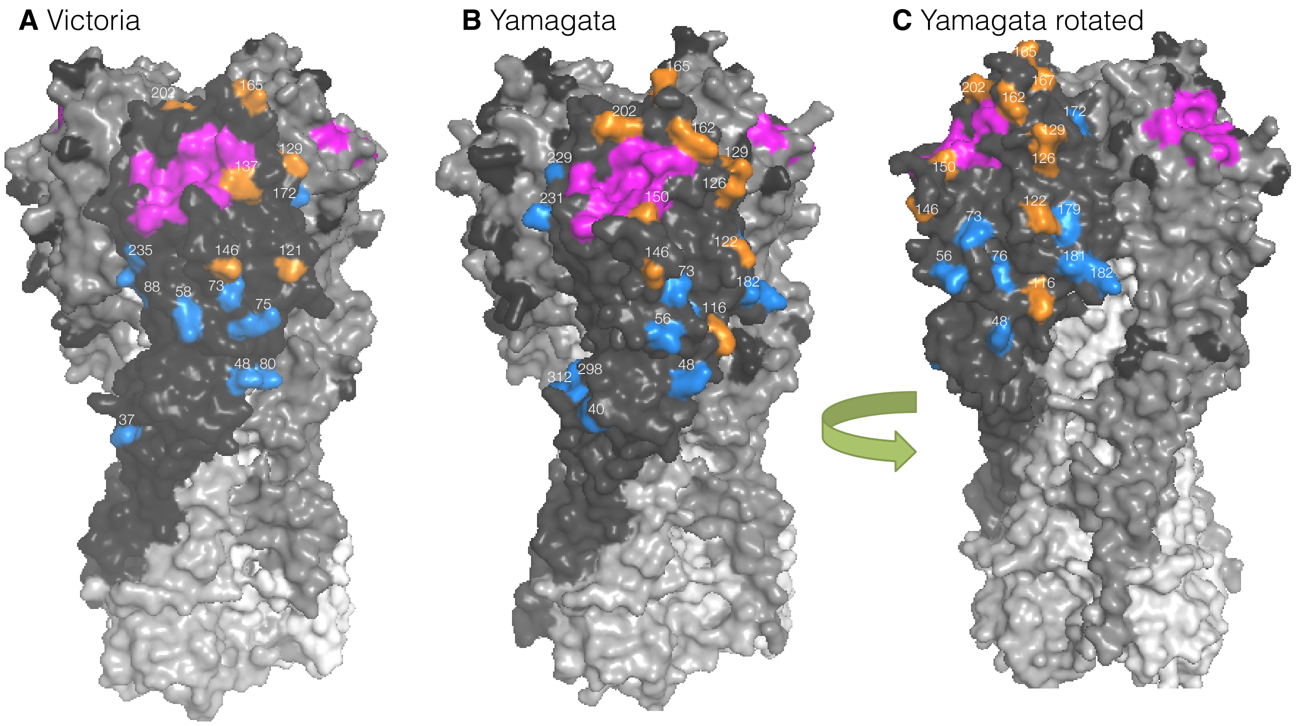

S7 Fig. Structural mapping of phylogenetic 'trunk' amino acid substitutions on Victoria-lineage and Yamagata-lineage HA trimers. HA1 units are in darker grey while HA2 are white-grey, with the RBS shown in pink. (A) Victoria-lineage virus B/Brisbane/60/2008 (PDB: 4FQM), (B) Yamagata-lineage virus B/Yamanashi/166/98 (PDB: 4M40) and (C) rotated Yamagata-lineage virus B/Yamanashi/166/98. On the front-facing selected HA1 monomer (darkest grey), trunk mutations are labeled with residue number and coloured by location: in antigenic epitope (orange) or other (blue), while they are shown in black on the other two monomers for simplicity.
