# Supplementary material for: Genome-wide evolutionary dynamics of influenza B viruses on a global scale
Source: PLoS Pathog. 2017 Dec 28;13(12):e1006749. doi: 10.1371/journal.ppat.1006749 (PMC5790164; doi:10.1371/journal.ppat.1006749)
Supplement: S8 Fig — Alignment was performed with Multalin (Corpet 1988) and plotted with ESPRIPT (Gouet et al. 1999). Secondary structure elements were assigned using the crystal structure of hemagglutinin influenza virus B/ Yamanashi/166/1998 in complex with avian-like receptor LSTa (PDB accession number 4M44) (Ni et al. 2013). Secondary structure elements are shown with an arrow and helices are shown as spirals. Residues which are highlighted red are fully conserved, residues which are colored red are partially conserved, and residues which are black are not conserved. Residues which are solvent accessible (as determined by ESPRIPT) are highlighted by black (fully exposed) and gray (partially exposed) bars below the sequence. Residues located at the receptor binding site were determined using the PISA EBI server (Krissinel and Henrick 2007) and are annotated with pink bars below the sequence. An asterisk is placed at positions at sites which do not map on or nearby the major epitopes. Four previously described (Wang et al. 2008) major epitopes on the Influenza B virus are annotated below the sequence with orange bars. Residues in close- proximity to these regions which undergo frequent amino-acid substitutions in influenza B virus HA (Ni et al. 2013; Wang et al. 2008; Nunes et al. 2008; Pechirra et al. 2005; Shen et al. 2009) are annotated with green bars. (PDF) [file ppat.1006749.s008.pdf]

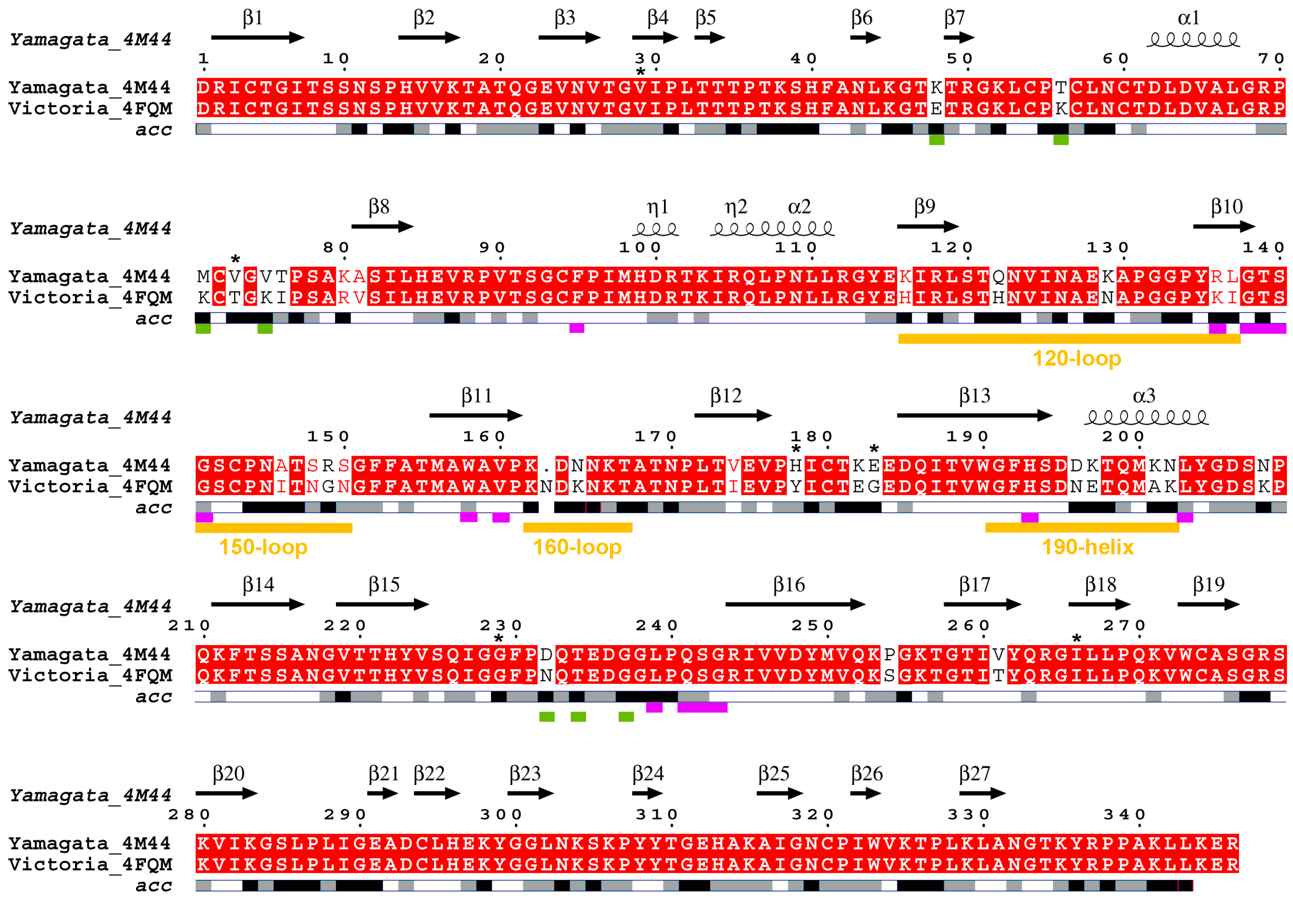

S8 Fig. Structure-based sequence alignment of influenza B/Yamagata and B/Victoria HA1. Alignment was performed with Multalin (Corpet 1988) and plotted with ESPRIPT (Gouet et al. 1999). Secondary structure elements were assigned using the crystal structure of hemagglutinin influenza virus B/Yamanashi/166/1998 in complex with avian-like receptor LSTa (PDB accession number 4M44) (Ni et al. 2013). Secondary structure elements are shown with an arrow and helices are shown as spirals. Residues which are highlighted red are fully conserved, residues which are colored red are partially conserved, and residues which are black are not conserved. Residues which are solvent accessible (as determined by ESPRIPT) are highlighted by black (fully exposed) and gray (partially exposed) bars below the sequence. Residues located at the receptor binding site were determined using the PISA EBI server (Krissinel and Henrick 2007) and are annotated with pink bars below the sequence. An asterisk is placed at positions at sites which do not map on or nearby the major epitopes. Four previously described (Wang et al. 2008) major epitopes on the Influenza B virus are annotated below the sequence with orange bars. Residues in close-proximity to these regions which undergo frequent amino-acid substitutions in influenza B virus HA (Ni et al. 2013; Wang et al. 2008; Nunes et al. 2008; Pechirra et al. 2005; Shen et al. 2009) are annotated with green bars.
