# Supplementary material for: Genome-wide evolutionary dynamics of influenza B viruses on a global scale
Source: PLoS Pathog. 2017 Dec 28;13(12):e1006749. doi: 10.1371/journal.ppat.1006749 (PMC5790164; doi:10.1371/journal.ppat.1006749)
Supplement: S9 Fig — Substitutions not located at potential PB1/PA or PB1/vRNA interface regions are highlighted as spheres coloured by emergence in Yamagata-lineage clade 2 (orange) or clade 3 (red) viruses. See Fig 6 legend for details. (PDF) [file ppat.1006749.s009.pdf]

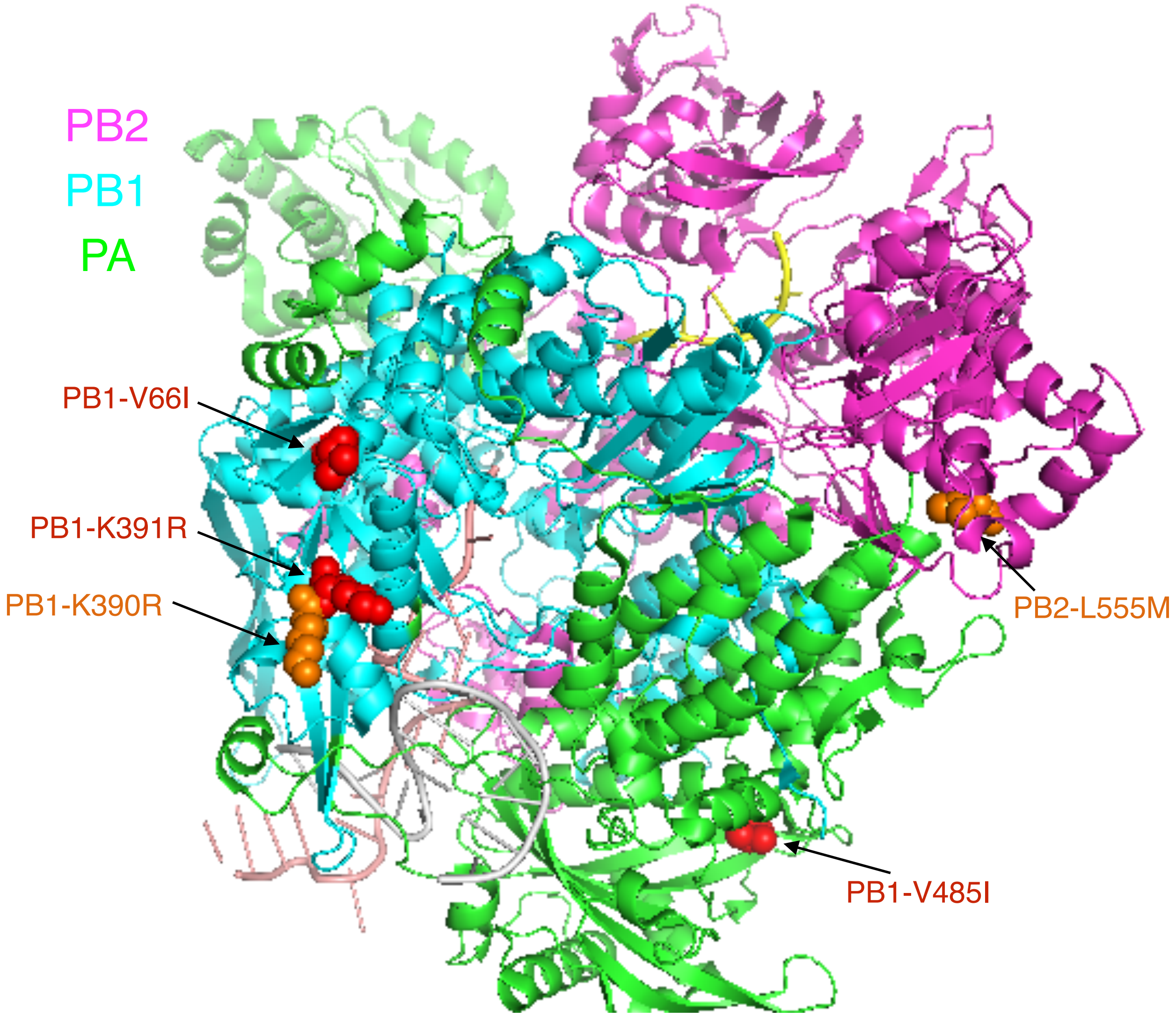

S9 Fig. Structural mapping of additional Yamagata-lineage 'clade-defining' trunk substitutions on influenza B polymerase complex. Substitutions not located at potential PB1/PA or PB1/vRNA interface regions are highlighted as spheres coloured by emergence in Yamagata-lineage clade 2 (orange) or clade 3 (red) viruses. See Fig 6 legend for details.
