# Supplementary material for: Genome-wide evolutionary dynamics of influenza B viruses on a global scale
Source: PLoS Pathog. 2017 Dec 28;13(12):e1006749. doi: 10.1371/journal.ppat.1006749 (PMC5790164; doi:10.1371/journal.ppat.1006749)
Supplement: S10 Fig — Clades are highlighted in colored blocks: Yamagata-lineage B/Florida/4/2006 (FR06) clade shown in yellow. See Fig 8 legend for other details. (PDF) [file ppat.1006749.s010.pdf]

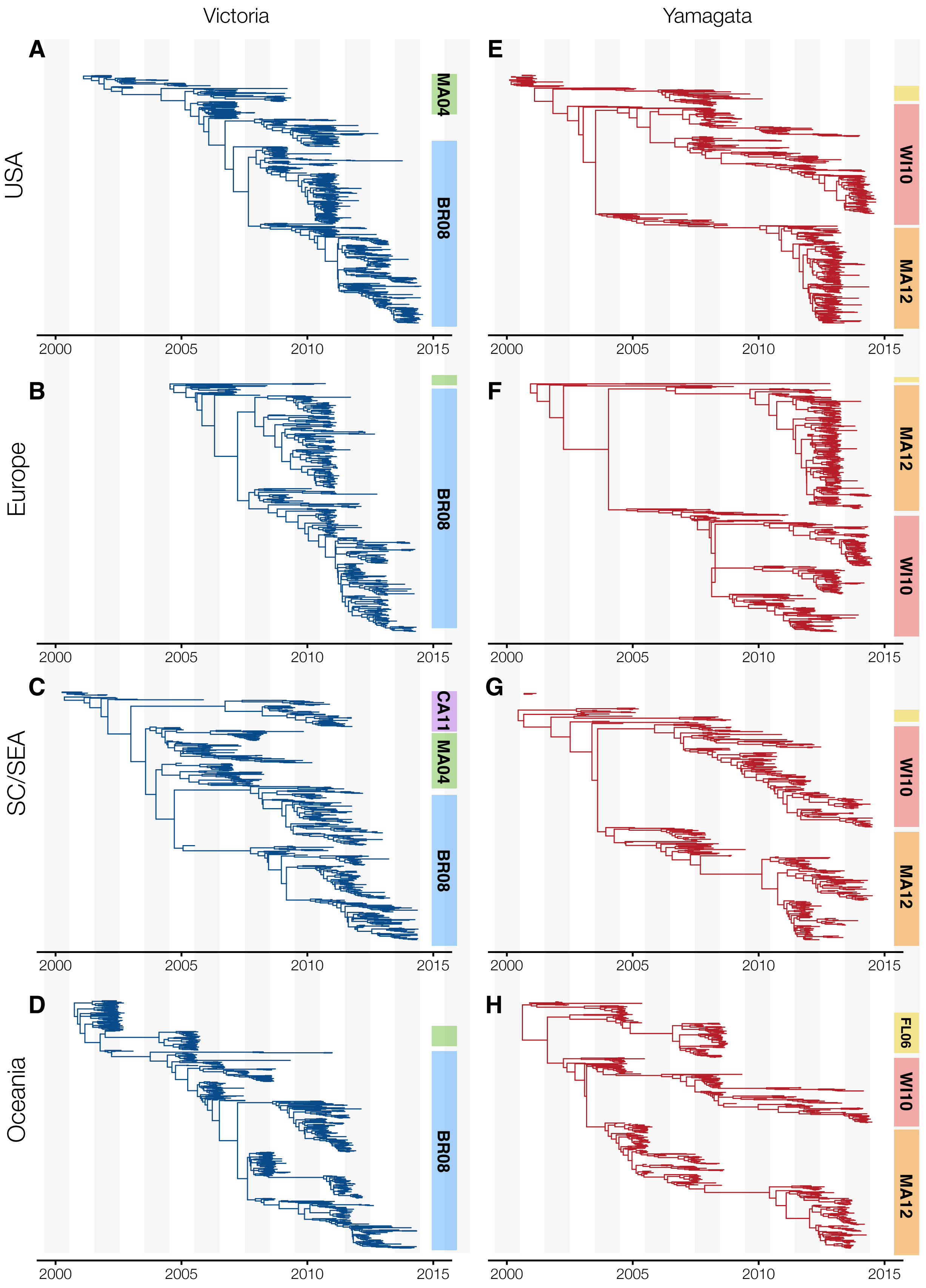

S10 Fig. Time-resolved HA gene phylogenies of influenza B viruses isolated in four major global regions from 2001-2014. Maximum-clade credibility (MCC) trees are shown for Victoria- (blue) and Yamagata-lineages (red) circulating in (A, E) USA, (B, F) Europe, (C, G) Southern China and Southeast Asia, and (D, H) Oceania. Clades are highlighted in colored blocks: Yamagata-lineage B/Florida/4/2006 (FR06) clade shown in yellow. See Fig 8 legend for other details.
