# Supplementary material for: Genome-wide evolutionary dynamics of influenza B viruses on a global scale
Source: PLoS Pathog. 2017 Dec 28;13(12):e1006749. doi: 10.1371/journal.ppat.1006749 (PMC5790164; doi:10.1371/journal.ppat.1006749)
Supplement: S11 Fig — Effective population sizes over time inferred by Bayesian skyride analysis for HA of Victoria- (blue) and Yamagata-lineage (red) viruses isolated in (A) the USA, (B) Europe, (C) SC/SEA and (D) Oceania. Solid lines represent median values and shaded areas represent 95% highest probability densities (HPD) credible intervals across MCMC samples. (PDF) [file ppat.1006749.s011.pdf]

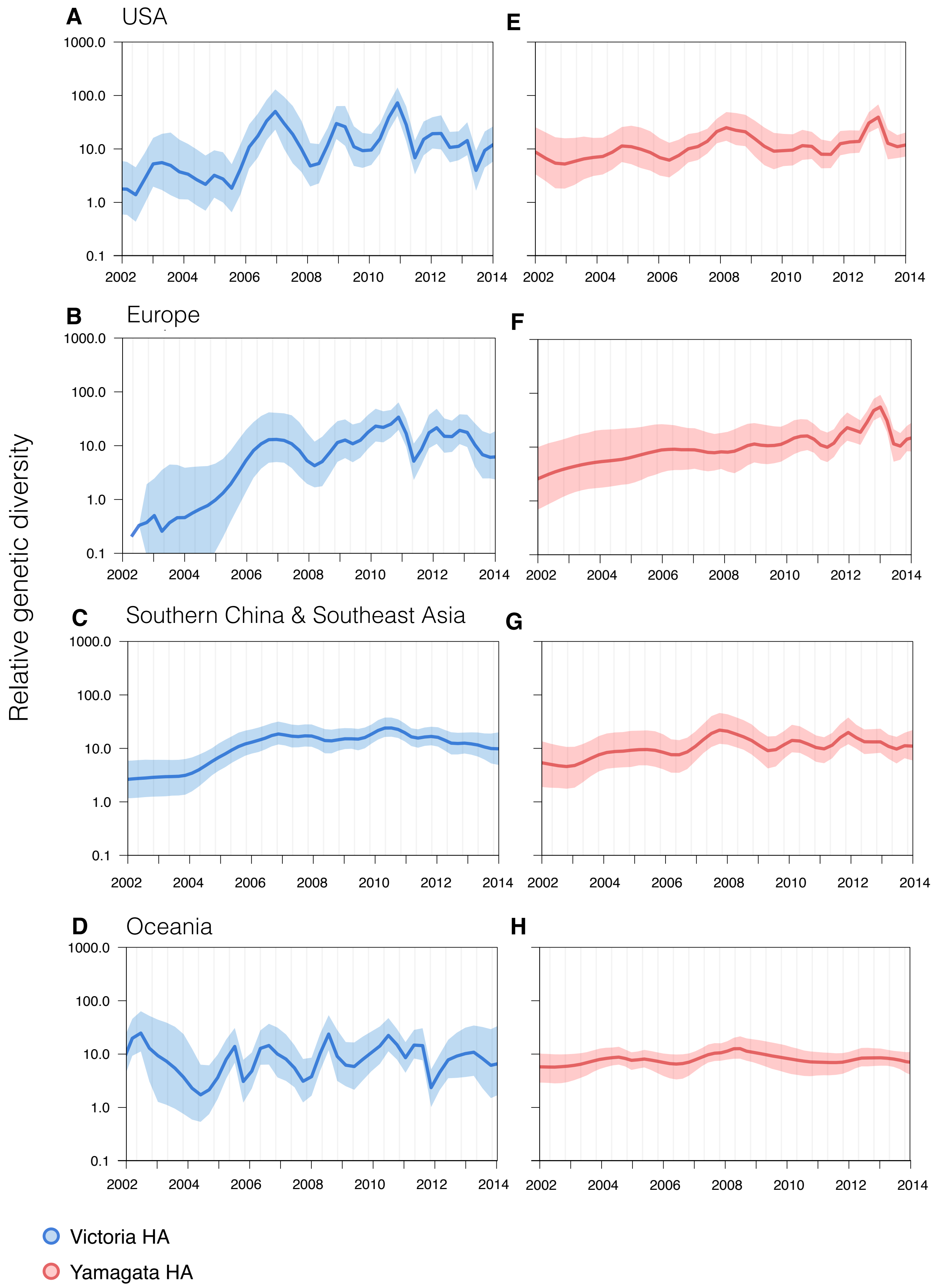

S11 Fig. Relative genetic diversity of HA genes of influenza B viruses circulating in different regions of the world. Effective population sizes over time inferred by Bayesian skyride analysis for HA of Victoria- (blue) and Yamagata-lineage (red) viruses isolated in (A, E) the USA, (B, F) Europe, (C, G) SC/SEA and (D, H) Oceania. Solid lines represent median values and shaded areas represent 95% highest probability densities (HPD) credible intervals across MCMC samples.
