# Supplementary material for: Genome-wide evolutionary dynamics of influenza B viruses on a global scale
Source: PLoS Pathog. 2017 Dec 28;13(12):e1006749. doi: 10.1371/journal.ppat.1006749 (PMC5790164; doi:10.1371/journal.ppat.1006749)
Supplement: S12 Fig — Time series of mean pairwise diversity for viruses collected from countries of the African Region of WHO (AFRO) and Eastern Mediterranean Region of WHO (EMRO) as listed on http://www.who.int/influenza/gisrs_laboratory/national_influenza_centres/list/en/ (Accessed 8 August 2016). Due to limited sampling, these regions are not discussed in the main manuscript. See Fig 6 legend for further details. (PDF) [file ppat.1006749.s012.pdf]

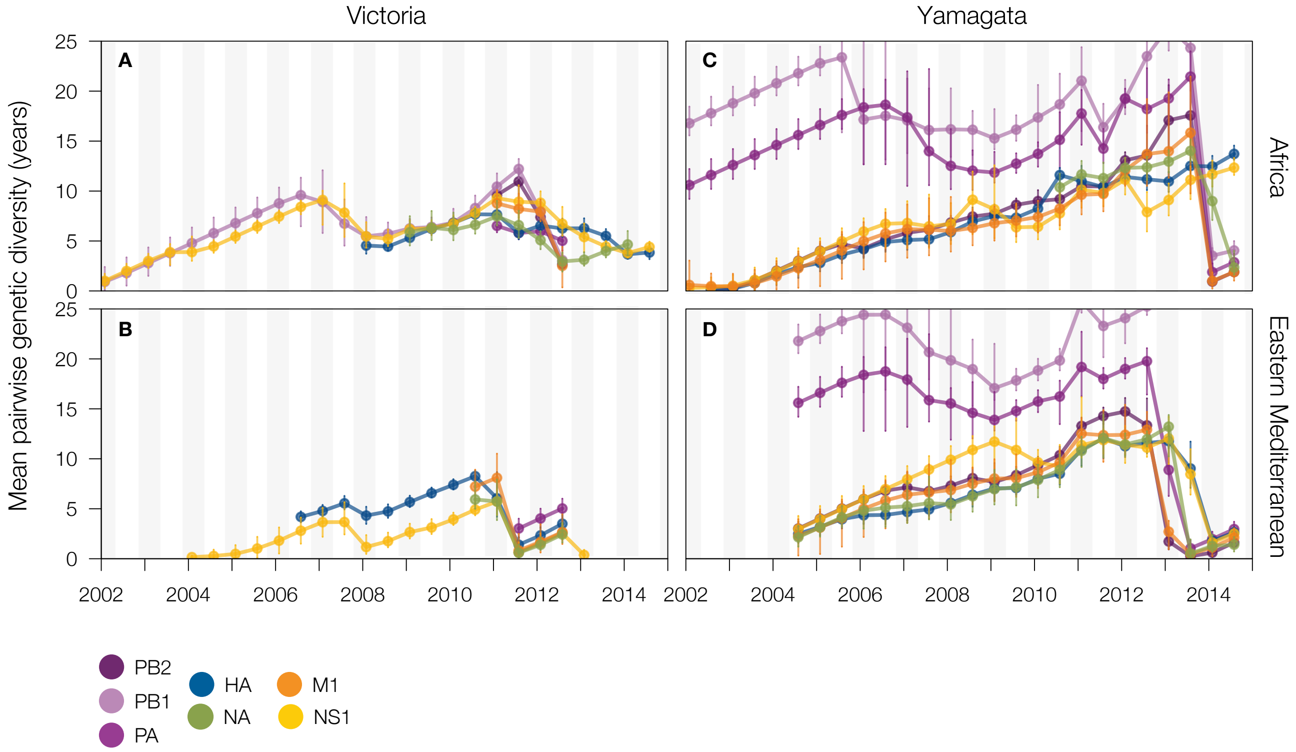

S12 Fig. Genetic diversity of gene segments over time in less-sampled geographic regions. Time series of mean pairwise diversity for Victoria and Yamagata lineage viruses collected from countries of the (A, C) African Region of WHO (AFRO) and (B, D) Eastern Mediterranean Region of WHO (EMRO) as listed on http://www.who.int/influenza/gisrs_laboratory/national_influenza_centres/list/en/ (Accessed 8 August 2016). Due to limited sampling, these regions are not discussed in the main manuscript. See Fig 6 legend for further details.
