# Supplementary material for: Genome-wide evolutionary dynamics of influenza B viruses on a global scale
Source: PLoS Pathog. 2017 Dec 28;13(12):e1006749. doi: 10.1371/journal.ppat.1006749 (PMC5790164; doi:10.1371/journal.ppat.1006749)
Supplement: S13 Fig — Due to limited sampling, these regions are not discussed in the main text. See Fig 5 legend for further details. (PDF) [file ppat.1006749.s013.pdf]

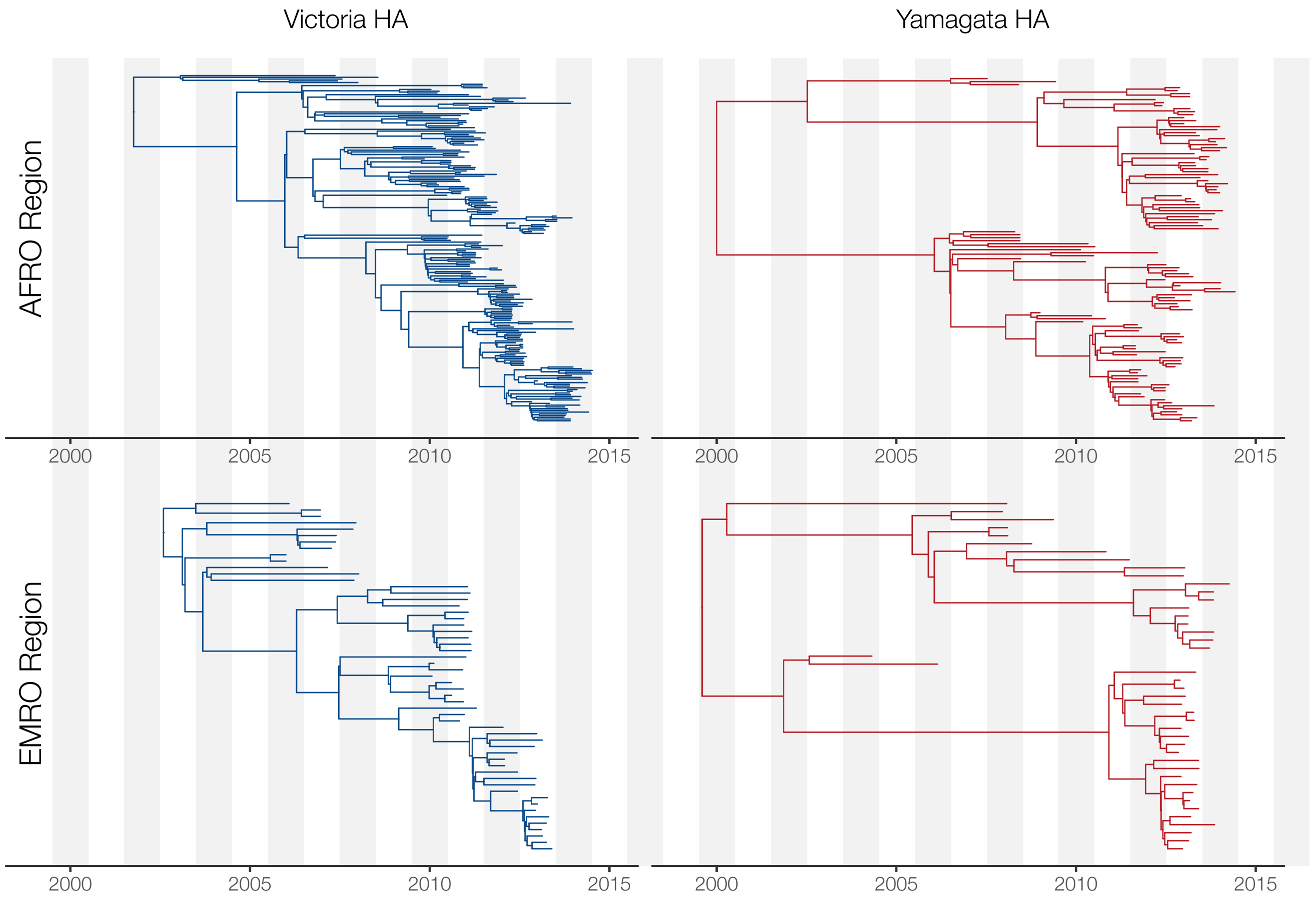

S13 Fig. Time-resolved HA and NA gene phylogenies of influenza B viruses isolated in WHO AFRO and EMRO regions from 2001-2014. Due to limited sampling, these regions are not discussed in the main text. See Fig 5 legend for further details.
