# Supplementary material for: Genome-wide evolutionary dynamics of influenza B viruses on a global scale
Source: PLoS Pathog. 2017 Dec 28;13(12):e1006749. doi: 10.1371/journal.ppat.1006749 (PMC5790164; doi:10.1371/journal.ppat.1006749)
Supplement: S1 Fig — Branches of phylogenies are labeled with amino acid substitutions occurring along the phylogenetic ‘trunk’ and are colored by well-supported clade distinctions. Nodes with greater than 0.70 posterior probability support are shown with circle node shapes. (PDF) [file ppat.1006749.s001.pdf]

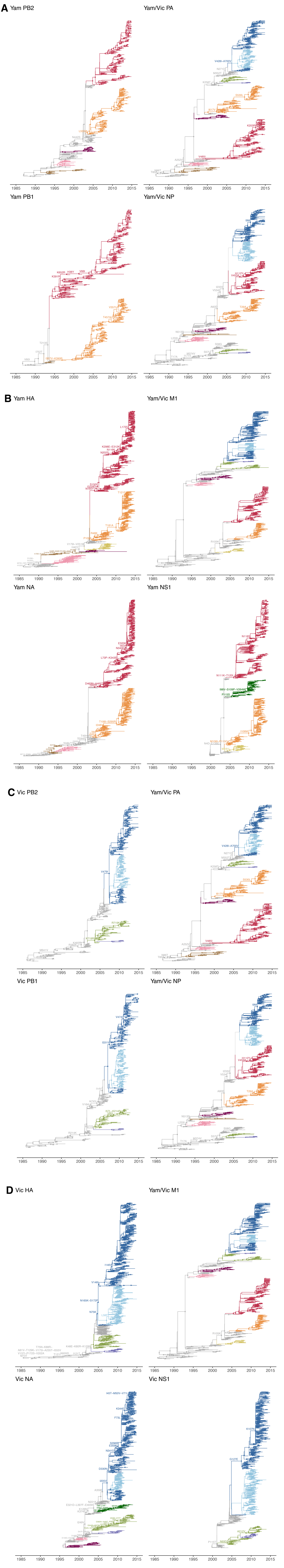

S1 Fig. Maximum-clade credibility trees for all major influenza B virus genes. Lineage-specific gene phylogenies shown for (A) Yamagata PB2, PB1, PA, NP, (B) Yamagata HA, NA, M1, NS1, (C) Victoria PB2, PB1, PA, NP, and (D) Victoria HA, NA, M1, NS1. Branches of phylogenies are labeled with amino acid substitutions occurring along the phylogenetic 'trunk' and are colored by well-supported clade distinctions. Nodes with greater than 0.70 posterior probability support are shown with circle node shapes.
